# CMTM7 inhibits breast cancer progression by regulating Wnt/β-catenin signaling

**DOI:** 10.1186/s13058-023-01620-9

**Published:** 2023-02-24

**Authors:** Zhao-Hui Chen, Yao Tian, Guang-Lei Zhou, Hao-Ran Yue, Xue-Jie Zhou, Hai-Yan Ma, Jie Ge, Xin Wang, Xu-Chen Cao, Yue Yu

**Affiliations:** 1 The First Department of Breast Cancer, Tianjin Medical University Cancer Institute and Hospital, National Clinical Research Center for Cancer, Huan-Hu-Xi Road, He-Xi District, Tianjin, 300060 China; 2 Key Laboratory of Breast Cancer Prevention and Therapy, Tianjin Medical University, Ministry of Education, Tianjin, 300060 China; 3 Key Laboratory of Cancer Prevention and Therapy, Tianjin, 300060 China; 4 Tianjin’s Clinical Research Center for Cancer, Tianjin, 300060 China; 5 Department of General Surgery, Tianjin Medical University General Hospital, Tianjin, 300052 China

**Keywords:** Breast cancer, CMTM7, miR-182-5p, CTNNA1, Wnt/β-catenin signaling

## Abstract

**Background:**

Breast cancer is the major cause of death in females globally. Chemokine-like factor like MARVEL transmembrane domain containing 7 (CMTM7) is reported as a tumor suppressor and is involved in epidermal growth factor receptor degradation and PI3K/AKT signaling in previous studies. However, other molecular mechanisms of CMTM7 remain unclear.

**Methods:**

The expression level of CMTM7 in breast cancer cells and tissues was detected by qRT-PCR and western blot, and the methylation of CMTM7 promoter was detected by BSP sequencing. The effect of CMTM7 was verified both in vitro and in vivo, including MTT, colony formation, EdU assay, transwell assay and wound healing assay. The interaction between CMTM7 and CTNNA1 was investigated by co-IP assay. The regulation of miR-182-5p on CMTM7 and TCF3 on miR-182-5p was detected by luciferase reporter assay and ChIP analysis.

**Results:**

This study detected the hypermethylation levels of the CMTM7 promoter region in breast cancer tissues and cell lines. CMTM7 was performed as a tumor suppressor both in vitro and in vivo. Furthermore, CMTM7 was a direct miR-182-5p target. Besides, we found that CMTM7 could interact with Catenin Alpha 1 (CTNNA1) and regulate Wnt/β-catenin signaling. Finally, transcription factor 3 (TCF3) can regulate miR-182-5p. We identified a feedback loop with the composition of miR-182-5p, CMTM7, CTNNA1, CTNNB1 (β-catenin), and TCF3, which play essential roles in breast cancer progression.

**Conclusion:**

These findings reveal the emerging character of CMTM7 in Wnt/β-catenin signaling and bring new sights of gene interaction. CMTM7 and other elements in the feedback loop may serve as emerging targets for breast cancer therapy.

**Supplementary Information:**

The online version contains supplementary material available at 10.1186/s13058-023-01620-9.

## Introduction

Breast cancer remains the prevalent malignancy and major cause of death in females worldwide [1]. Breast cancer patient prognosis has been greatly improved with advances in cancer therapy, but it is still incompletely satisfactory [2–4]. Breast cancer is a heterogeneous disease influenced by epigenetic, genetic, and environmental factors [5]. Recently, studies on the mechanism of breast cancer have made huge achievements, revealing the initiation and progression of breast cancer [6–8]. However, the carcinogenic pathways and novel pathogenic genes still need further investigation.

Chemokine-like factor (CKLF)-like MARVEL transmembrane domain containing 7 (CMTM7) is located on the chromosome 3p22.3 and serves as a member of the CMTM family (CMTMs), which includes CMTM1-8 and CKLF in human [9]. CMTMs are involved in the structural and functional intermediation between the transmembrane-4 superfamily and classical chemokines [9–11]. CMTM7 serves as a tumor inhibitor in multi-cancer types. Liu et al. reported that CMTM7 interacts with ATG14L, Beclin1, VPS34 and Rab5, thereby facilitating autophagosome formation [12]. Besides, CMTM7 can inhibit the growth of esophageal squamous cell carcinoma (ESCC) and non-small cell lung cancer (NSCLC) by suppressing EGFR/AKT signaling [13, 14]. Furthermore, Xiao et al. highlighted the importance of CMTM7 in epithelial-mesenchymal transition (EMT)-induced PD-L1 [15]. These findings provide convincing evidence that CMTM7 plays a critical role in cancer. However, the underlying mechanism of CMTM7 in breast cancer still needs further investigation.

Abnormal regulation of the Wnt signaling is a prevalent issue in the cancer process. The Wnt signaling is subdivided into two categories: canonical and noncanonical [16]. Canonical Wnt signaling, which is also known as Wnt/β-catenin signaling, is tightly associated with CTNNB1 subsequent nuclear translocation and stabilization, which can activate β-catenin-TCF/LEF target genes transcriptionally, such as CCND1 (Cyclin D1), MYC (c-myc), and SNAI1 [17]. While Wnt-planar cell polarity (PCP) signaling, which is a noncanonical Wnt signaling, does not require β-catenin or TCF molecules. Besides, Wnt-Ca^2+^ signaling is an another noncanonical Wnt signaling with less research, but performs crucial roles in multiple biology process [18]. In recent years, studies have uncovered that Wnt signaling is involved in proliferation [19], metastasis [20, 21], stemness maintenance [22], phenotype shaping [23], immune microenvironment regulation [24], and therapeutic resistance [25] in breast cancer. Therefore, it is essential to investigate the molecular mechanism of Wnt signaling for breast cancer therapy.

This study aims to reveal CMTM7 as the direct target of miR-182-5p and could interact with CTNNA1 and inhibit the nuclear localization of CTNNB1, downregulating Wnt signaling and its target genes. Furthermore, TCF3 could transcriptionally activate miR-182-5p, inhibiting the CMTM7 expression. A feedback loop formed by miR-182-5p/CMTM7/CTNNA1/CTNNB1/TCF3 may play a critical role in breast cancer.

## Materials and methods

### Breast cancer specimen

Breast cancer (*n* = 189 cases) and paired adjacent normal tissues were gathered to represent the study sample. All patients attended Tianjin Medical University Cancer Institute and Hospital, and their diagnoses were confirmed histologically. The breast cancer tissues were collected immediately after mastectomies and followed by snap-frozen in liquid nitrogen. The tissues were stored at − 80 °C for the following analysis. All protocols were sanctioned by the Ethical Committee of Tianjin Medical University Cancer Institute and Hospital. Informed and written consent was acquired from all involved patients.

### Immunohistochemistry (IHC)

Heat-induced antigen retrieval was conducted on deparaffinized, dehydrated tissue sections in 0.01 M sodium citrate buffer at 98 °C for 18 min. The 3% H_2_O_2_ was utilized to block endogenous peroxidase activity. Then, the sections were treated with 5% bovine serum albumin (BSA) for 1 h at room temperature (RT) and treated with primary antibodies at 4 °C overnight. The sections were washed with phosphate buffer saline (PBS), treated with HRP-labeled secondary antibodies for 1 h at RT, and then visualized with 3,3′-diaminobenzidine (DAB) reagent. Eventually, the sections were covered with neutral gum after hematoxylin staining and hydrochloric acid alcohol differentiation. All sections were captured and estimated by two professionals under a light microscope.

### Cell culture, plasmids, and transfection

Normal breast epithelial cell line MCF-10A, breast cancer cell lines MDA-MB-231, MCF-7, SK-BR3, T47D, and Cal51 were obtained from ATCC (American Type Culture Collection, Manassas, USA). MCF-10A was cultured in MCF-10A cell-specific medium (Procell, China). MCF-7, T47D and MDA-MB-231 were cultured in DMEM (Gibco, USA), while Cal51 in 1640 (Gibco, USA). All medium included 10% fetal bovine serum (FBS, NEWZERUM, Australia) and 1% penicillin/streptomycin (Gibco, USA). The cells were kept at 37 °C in a humidified 5% CO_2_ atmosphere cell incubator.

The CMTM7 and TCF3 plasmids and their corresponding vectors, as well as miR-182-5p mimic, inhibitor, and the siRNA kits of CMTM7, were obtained from RiboBio (Guangzhou, China). The miRNAs and siRNAs oligonucleotides are listed in Additional file 1: Table S1. The CMTM7 3′UTR region, the miR-182-5p promoter region, and their mutants were cloned into the pGL4.17-basic vector (Promega, USA). The transfection was conducted utilizing Lipofectamine 3000 (Invitrogen, USA) in accordance with the manufacturer’s recommendations. Lentiviruses (RiboBio, China) were utilized to infect MCF-7 cells and generate stable CMTM7 overexpressed cells by the manufacturer’s protocol.

### Antibodies and reagents

Antibodies against CMTM7 (ID: PA5-103744, Invitrogen, USA), Flag (ID: YM3001, Immunoway, China), IgG (ID: RS0002, Immunoway, China), E-cadherin (ID: 14472), N-cadherin (ID: 13116), vimentin (ID: 5741), cyclinD1 (ID: 55506), c-myc (ID: 18583), Survivin (ID: 2808), β-catenin (ID: 8480), α-catenin (ID: 2131) and β-actin (ID: 3700, Cell Signaling Technology, USA) were used. Reagents of decitabine (5-Aza-2′-deoxycytidine, MCE, USA), Adavivint (SM04690, GLPBIO, USA), and BML-284 (Wnt agonist 1, Adooq, USA) were used.

### RNA isolation and quantitative reverse transcription PCR (RT-qPCR)

Total RNA was isolated from tissues and cells using Trizol reagent (Invitrogen, USA) based on the manufacturer’s recommendations. RNA quality and concentration were measured using NanoDrop 2000 spectrophotometer (Thermo Scientific, USA). For mRNA, All-in-One First-Strand cDNA Synthesis SuperMix for qPCR (Transgene, China) was used for RT-qPCR, while for miRNA, the miRNA RT-qPCR Starter kit (RiboBio, China) was used. The CMTM7 and GAPDH primers were synthesized by Genewiz (Tianjin, China), and miR-182-5p (ID: 002334) and U6 (ID: 001093) probes were purchased from Thermo Fisher Scientific (USA). All specific sequences are listed in Additional file 1: Table S2.

### DNA isolation and bisulfite sequencing PCR (BSP)

Genomic DNA was isolated from cells and tissues utilizing a genomic DNA isolation kit (Thermo Scientific, USA). The EpiTect bisulfite kit (Qiagen, USA) was utilized for DNA conversion. The bisulfite-converted sequences of the CMTM7 promoter region (− 229 to + 171) were amplified. Products were retrieved and purified utilizing a EasyPure Quick Gel Extraction Kit (Transgene, China), and then inserted into a pGEM-T easy vector system (Promega, USA). Ten colonies from each ligation were randomly picked and sequenced. The methylation status was estimated based on the presence of C (methylated) or T (non-methylated). The primers used for BSP are showed in Additional file 1: Table S3.

### Proliferation assay

For MTT assay, cells were planted into 96-well plates at a density of 2 × 10^3^ cells per well, and 20 µL MTT solution (5 mg/mL) was added on days of 1, 2, 3, 4, and 5. After 4 h of dark incubation, the cells were starved and treated with dimethyl sulfoxide (DMSO). The absorbance values were detected and analyzed using a microplate reader (Bio‐Rad, USA) at 570 and 630 nm.

For colony formation, 5 × 10^2^ cells were planted into six-well plates and incubated for a period of time until the colonies reached the appropriate size. Then, the colonies were fixed with 4% paraformaldehyde and stained with 1% crystal violet, followed by image acquisition and count.

EdU assay was conducted utilizing EdU Assay Kit (RiboBio, China) under manufacturers’ instructions. The EdU-positive cell ratio was counted and analyzed using a fluorescence microscope (Zeiss, Germany).

### Migration and invasion assay

The Matrigel-coated Transwell (BD Biosciences, USA) was used to detect breast cancer cell invasive abilities. Briefly, 6 × 10^4^ cells were seeded into upper chambers with FBS-free medium, while medium contained 20% FBS was added in the lower chambers. After 14–36 h incubation, the migration cells were fixed and stained using a three-step set (Thermo Scientific, USA). Then, the images were screened and counted under a light microscope (Olympus, Japan) at 100 × magnification.

For cell scratch assay, the cells were incubated in 6-well plates for 48 h after transfection to reach confluency. A sterile 10 μL pipette tip was used to make a single scratch in each well. All cells were then incubated in an FBS-free medium to exclude the FBS effect on migration. The images of the scratches were captured at the time of 0 and 24 h under a light microscope (Olympus, Japan). The distances (ratio to 0 h) were measured and analyzed.

For single-cell time-lapse imaging assay, the cells were seeded in 24-well plates and scanned by a high intension imaging system (PerkinElmer, PE operetta CLS, USA). The migration of cells was captured once an hour (total 14 h). The trajectory of the cell was auto-identified, and the migration speed was analyzed.

### Flow cytometry assay

For the cell cycle assay, the breast cancer cells were digested with 0.25% trypsin and washed 3 times with PBS, after which, the cells were fixed in 95% ethanol at − 20 °C overnight. Before Onboard detection, PI staining solution (Cell Signaling Technology, USA) containing 50 μg/mL PI and 100 μg/mL RNase A was used following the manufactures’ recommendation. For apoptosis assay, the cells were stained utilizing Annexin V Apoptosis Detection Kit (BD Pharmingen, USA) in accordance with the official instruction after digestion and washing. A flow cytometer (BD Biosciences, USA) was used for analysis.

### Western blotting and immunofluorescence (IF)

The SDS-PAGE electrophoresis was conducted to separate cell lysates. The target proteins were immunoblotted with corresponding antibodies and visualized with an ECL reagent (Millipore, Bedford, MA, USA). The scale values of each single stripe were measured by ImageJ software and normalized by β-actin (Additional file 1: Fig. S1). For the immunofluorescence assay, cells were planted onto glass coverslips at 3 × 10^4^ cells per well, followed by washing, fixation, and permeabilization. Primary antibodies were incubated on coverslips overnight at 4 °C. Then, the coverslips were treated with FITC-/TRITC-conjugated secondary antibodies for 1 h at RT. After being stained with 4′,6-diamidino-2-phenylindole (DAPI), the coverslips were captured and analyzed using a fluorescence microscope (Zeiss).

### Co-immunoprecipitation (Co-IP) and protein identification

The breast cancer cells were washed with pre-cooled PBS and treated with IP lysis buffer (Thermo, USA). Then, the lysates were immunoprecipitated with specific antibodies targeting against CMTM7, Flag, α-catenin or IgG (negative control) at 4 °C overnight. The next day, pre-cleaned 40 μL Protein A/G Agarose Beads (SantaCruz, USA) were added into the system and incubated for 2 h at 4 °C. Then, the agarose beads were washed with PBS for 3 times, and the immunoprecipitation complexes were isolated. For Co-IP assay, the lysates were conducted with immunoblotting as described above. For protein identification, the lysates were stained with coomassie brilliant blue solution (Solarbio, China) after being separated on SDS-PAGE gels. The proteins were identified through mass spectrometry, which was performed and analyzed by APT biotech company (China).

### Chromatin immunoprecipitation (ChIP) analysis

ChIP assay was conducted as previously described according to the manufacturer’s recommendations of Upstate Biotechnology [26]. The involved primers are listed in Additional file 1: Table S3.

### Luciferase reporter and TOP/FOP assay

The promoter regions of CMTM7 and miR-182-5p were cloned into a pGL4.17-basic vector (Promega) and transfected into breast cancer cells along with their corresponding controls and PRL-TK plasmid (Promega). Similarly, TOP/FOP-Flash (Genechem) was co-transfected into cells with CMTM7 overexpression or silence vector. The Dual-Luciferase Reporter Assay Kit (Transgene) was utilized to detect Firefly and Renilla luciferase activities in accordance with instructions.

### Xenograft

Stable CMTM7-overexpressing MCF-7 cells and control cells (8 × 10^6^ cells) were injected with 5% Matrigel (BD Biosciences, USA) into the mammary fat pads of 5-week-old female server combined immune-deficiency (SCID) mice and raised for six weeks, during which the tumor growth was recorded once a week. After execution, the final volume and weight were determined. To estimate the metastasis ability, cells were injected intravenously. Bioluminescence imaging was conducted utilizing a Xenogen IVIS 200 Imaging System (Caliper Life Sciences, USA) at 7, 14, 21, 28, and 35 days to detect metastasis ability. The paraffin-embedded tumors, lungs and livers obtained from mice were sectioned for hematoxylin–eosin (H&E) staining and IHC analysis. Each single group contained six mice, and all protocols were approved by the Ethics Committee of Tianjin Medical University Cancer Institute and Hospital.

### Statistics and analysis

Prism 8 (Graph pad Software, CA) was utilized for data visualization and analysis. A Student’s *t* test was utilized for comparisons of two-group, while the correlation analysis was conducted by Pearson correlation analysis. The data were exhibited as the mean ± standard deviation (SD), and *p* < 0.05 was considered to be significant.

## Results

### CMTM7 was downregulated in breast cancer

**Fig. 1:**
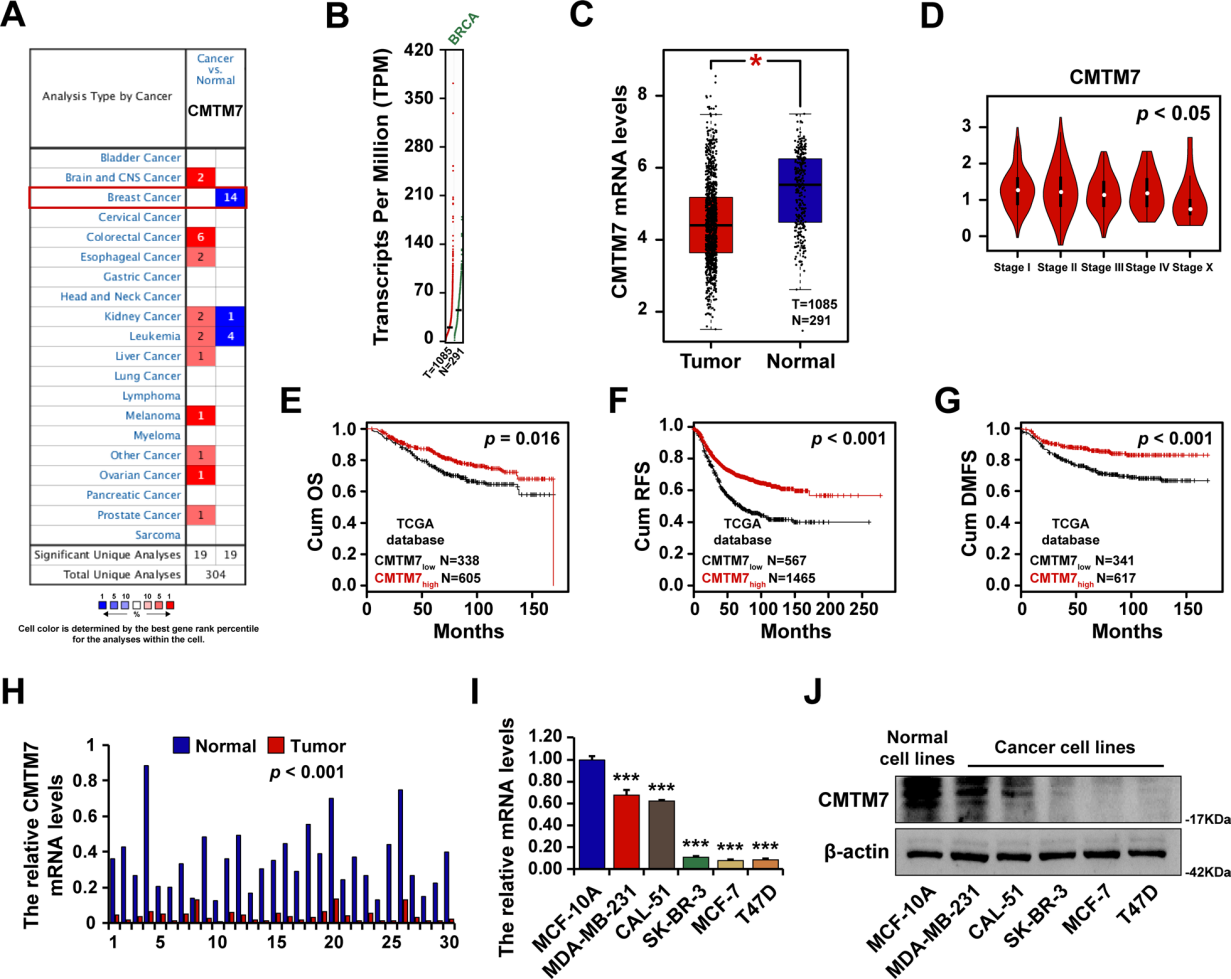
CMTM7 was downregulated in breast cancer. **A** The CMTM7 expression level in multiple cancer types. The CMTM7 expression level in breast cancer tissues compared with normal tissues (**B**, **C**). The CMTM7 expression level among breast cancer stages (**D**). The survival analysis of breast cancer patients with high- or low-CMTM7 expression levels, including cumulative OS (**E**), RFS (**F**), and DMFS (**G**), predicted by the KM plotter. **H** The CMTM7 mRNA expression level in 30 paired breast cancer tissues detected by RT-qPCR. The CMTM7 expression level in breast cancer cell lines detected by RT-qPCR (**I**) and western blot (**J**). *p < 0.05; ***p < 0.01

The expression levels of CMTM family members in multi-cancer types were estimated using Oncomine dataset (https://www.oncomine.org/), which showed their abnormal expression levels in different cancers (Additional file 1: Fig. S2A). Among all CMTM members, the CMTM7 expression was significantly lower in breast cancer (Fig. 1A). Then, the expression pattern and prognostic analysis of CMTMs were established through the Gene Expression Profiling Interactive Analysis (GEPIA) website (http://gepia.cancer-pku.cn/) (Additional file 1: Fig. S2B, C, D, and E). Similarly, CMTM7 had higher expression in normal breast tissues than in tumor tissues and related to earlier stages (Fig. 1B–D). Moreover, as predicted by Kaplan–Meier (KM)-plotter (http://kmplot.com/analysis), a higher CMTM7 expression level was associated with a better prognosis, no matter cumulative overall survival (OS) (Fig. 1E), recurrence-free survival (RFS) (Fig. 1F), or distant metastasis-free survival (DMFS) (Fig. 1G). The RT-qPCR analysis of CMTM7 mRNA expression levels in 30 paired clinical tissues revealed that breast cancer tissues had significantly lower CMTM7 mRNA levels than paired normal tissues (Fig. 1H). Finally, RT-qPCR (Fig. 1I) and western blot (Fig. 1J) were conducted to detect CMTM7 mRNA and protein levels in breast cell lines, including normal breast cell line MCF10A and breast cancer cell lines MDA-MB-231, CAL-51, SKBR3, MCF-7, and T47D, indicating that CMTM7 was decreased in all breast cancer cell lines as compared to MCF10A cells. Interestingly, the expression level of CMTM7 was much higher in triple-negative breast cancer cells MDA-MB-231 and CAL-51, compared with SKBR3, MCF7 and T47D cells. Taken together, these results showed that CMTM7 was downregulated in breast cancer tissues and cell lines, and the CMTM7 loss was related to a poor prognosis.

### DNA methylation of CMTM7 in breast cancer

**Fig. 2:**
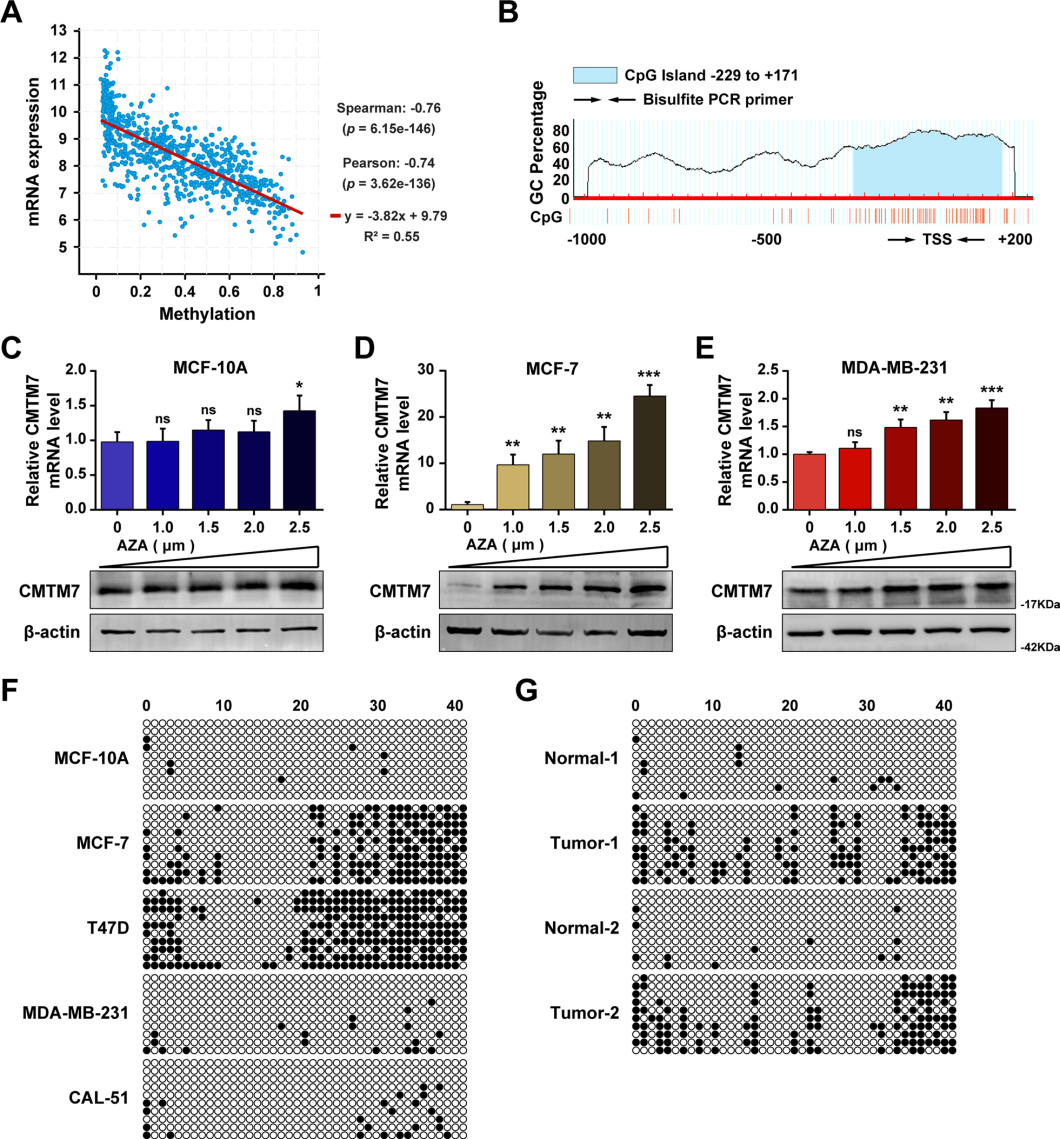
DNA methylation of CMTM7 in breast cancer. **A** The correlation between CMTM7 expression and DNA methylation. **B** The CpG island on the promoter region of CMTM7 predicted by the Meth primer website. The CMTM7 expression level detected by RT-qPCR and western blot after treatment of AZA as a dose escalation manner in MCF 10A cells (**C**), MCF-7 cells (**D**), and MDA-MB-231 cells (**E**). The methylation status of CMTM7 detected by BSP sequencing in breast cancer cell lines (**F**) and two paired breast cancer tissues (**G**). *p < 0.05; ***p < 0.01

A previous study reported a close association between CMTM7 gene expression and DNA methylation [13]. Herein, we analyzed the correlation between CMTMs and their promoters’ methylation status by cBioPortal (http://www.cbioportal.org/), which result showed that CMTM7 presented the most closed correlation with methylation regulation (Fig. 2A) compared to other CMTM family members (Additional file 1: Fig. S3). Besides, A CpG island was predicted to exist in the promoter of CMTM7 through the website of MethPrimer (http://www.urogene.org/methprimer/) (Fig. 2B). To further validate the methylated regulation of CMTM7 promoter region, the DNA methyltransferases (DNMT) inhibitor AZA was used for treating breast cancer cells at the concentrations of 1.0, 1.5, 2.0, and 2.5 µM. After four days of incubation, the CMTM7 expression level was measured using RT-qPCR and western blot, which results showed that the expression level of CMTM7 exhibited an AZA dose-dependent manner in highly methylated MCF-7 cells and MDA-MB-231 cells but was less significant in non-methylated MCF-10A cells (Fig. 2C–E). Furthermore, BSP sequencing was performed to detect the methylation status of CMTM7 promoter in breast cancer cell lines and tissues, and hypermethylation status of the CMTM7 promoter was detected in low CMTM7 mRNA-expressing breast cancer tissues and cell lines (Fig. 2F, G), indicating a negative association between the CMTM7 expression level and the methylation status of its CpG island in breast cancer.

### CMTM7 inhibited cell proliferation, invasion, and migration

**Fig. 3:**
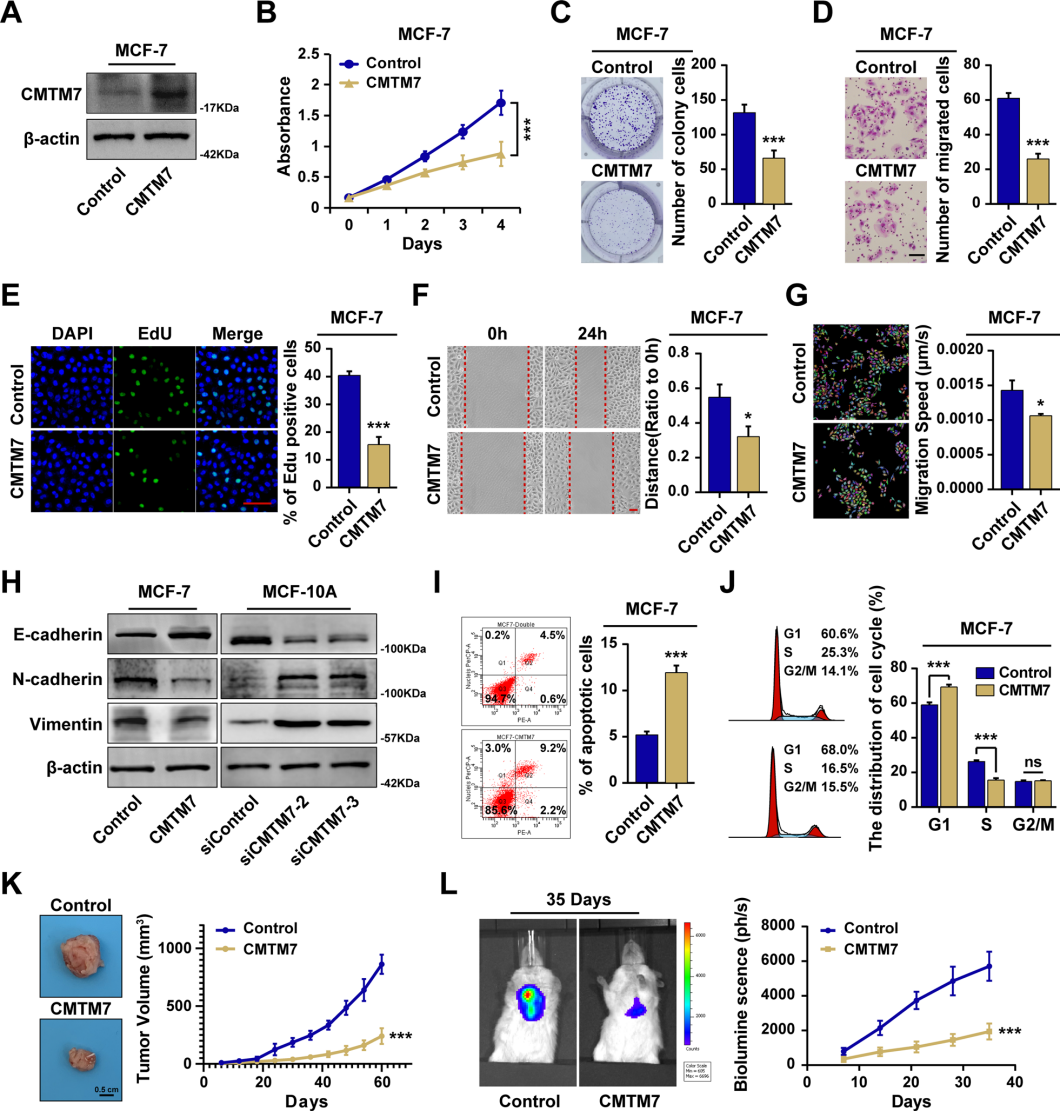
CMTM7 inhibited cell proliferation, migration, and invasion. **A** The construction of CMTM7-overexpressed MCF-7 cells validated by western blot. The cell proliferation assays were conducted, including MTT (**B**), colony formation assay (**C**), and EdU assay (**E**). The invasion and migration assays were performed, including transwell assay (**D**), scratch assay (**F**) and single-cell time-lapse imaging assay (**G**). **H** The expression levels of EMT-related biomarkers, including E-cadherin, N-cadherin, and Vimentin in CMTM7-overexpressed MCF-7 cells (left) and CMTM7-depleted MCF-10A cells (right) detected by western blot. The apoptotic cells (**I**) and the distribution of cell cycle (**J**) were analyzed through cell cytometry. **K** The image and growth curve of tumor volume after in situ injection in SCID mice. **L** The image of bioluminescence and curve after intravascular injection. *p < 0.05; ***p < 0.01

To investigate the effect of CMTM7 on breast cancer progression, the stable CMTM7-overexpressed MCF-7 cells were constructed and verified by western blot (Fig. 3A). CMTM7-overexpressed cells exhibited a lower proliferation rate (Fig. 3B), less colony formation (Fig. 3C), and a lower EdU positive ratio compared to control cells (Fig. 3E), indicating that CMTM7 could inhibit breast cancer cell proliferation. Meanwhile, CMTM7-overexpressed cells were less aggressive in the transwell (Fig. 3D) and scratch assays (Fig. 3F). Besides, the migration speed of CMTM7-overexpressed MCF-7 cells was slower than control group detected by single-cell time-lapse imaging assay (Fig. 3G, Additional file 2: Video 1, Additional file 3: Video 2). Moreover, the western blot showed that CMTM7-overexpressed MCF-7 cells exhibited more E-cadherin, less N-cadherin, and less Vimentin (Fig. 3H, left), while CMTM7-depleted MCF-10A cells were associated with less E-cadherin, more N-cadherin and more Vimentin (Fig. 3H, right), indicating that CMTM7 was negatively correlated with EMT. Additionally, the flow cytometry assay revealed that CMTM7 promoted cell apoptosis (Fig. 3I) and suppressed cell cycle progression (Fig. 3J). To evaluate the role of CMTM7 in vivo, the CMTM7-overexpressed MCF-7 cells were in situ injected or intravenously injected into SCID mice, which results showed that CMTM7 inhibited tumor growth (Fig. 3K) and metastasis (Fig. 3L). The similar results were obtained in CMTM7-overexpressed T47D cells (Additional file 1: Fig. S4), and cell functional experiments of siCMTM7 were performed in MDA-MB-231 and CAL-51 cells (Additional file 1: Fig. S5, Additional file 4: Video 3, Additional file 5: Video 4, Additional file 6: Video 5 and Additional file 1: Fig. S6). These results revealed that CMTM7 could inhibit breast cancer proliferation, invasion, and migration both in vitro and in vivo.

### CMTM7 interacted with CTNNA1

**Fig. 4:**
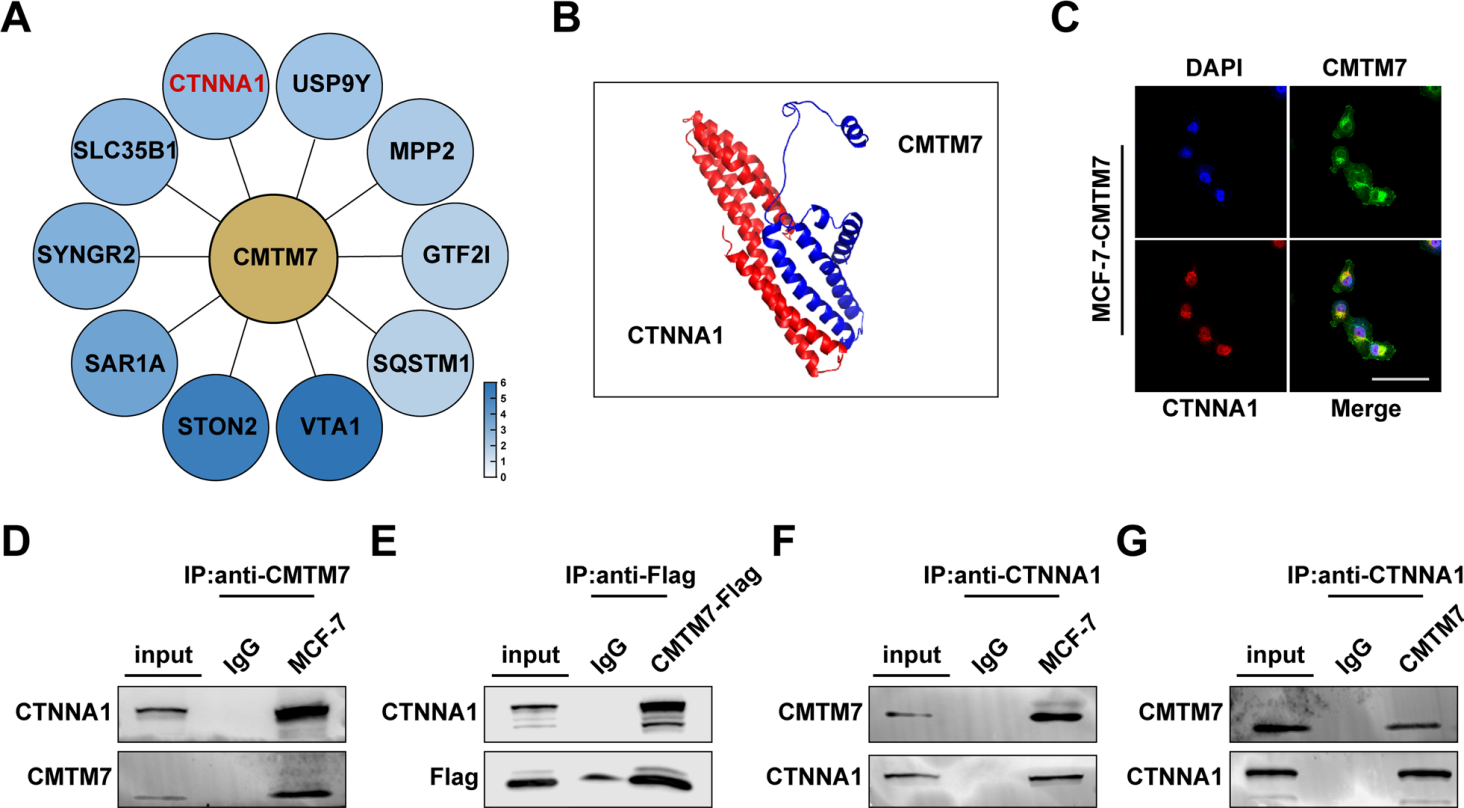
CMTM7 interacted with CTNNA1. **A** The top 10 interacting genes identified by mass spectrometry. **B** The predictive protein binding model of CMTM7 and CTNNA1. **C** The intracellular colocalization of CMTM7 and CTNNA1 visualized by IF. The interaction of CMTM7 and CTNNA1 was verified by co-IP assay, including endogenous (**D**, **F**) and exogenous (**E**, **G**). *p < 0.05; ***p < 0.01

Immunoprecipitation was performed and followed by mass spectrometry to further determine the mechanism of CMTM7 function. A total number of 649 CMTM7 binding proteins were identified, among which, CTNNA1 ranked at the leading and was selected as a cancer-related target gene (Fig. 4A). Then, the protein binding of CMTM7 and CTNNA1 was predicted, analyzed, and visualized by GRAMM-X (Protein–Protein Docking Web Server v.1.2.0), PDBePISA (https://www.ebi.ac.uk/) and PyMOL2 software (v.2.5), respectively, and the binding model of CMTM7 and CTNNA1 was shown as cartoon structures (Fig. 4B). Besides, the co-localization of CMTM7 and CTNNA1 in breast cancer cells was verified using immunofluorescence (Fig. 4C). Furthermore, the interaction between CMTM7 and CTNNA1 was confirmed by endogenous and exogenous co-immunoprecipitation (Fig. 4D–G). These findings demonstrated that CMTM7 could bind and directly interact with CTNNA1.

### CMTM7 suppressed Wnt signaling by suppressing the nuclear localization of β-catenin

**Fig. 5:**
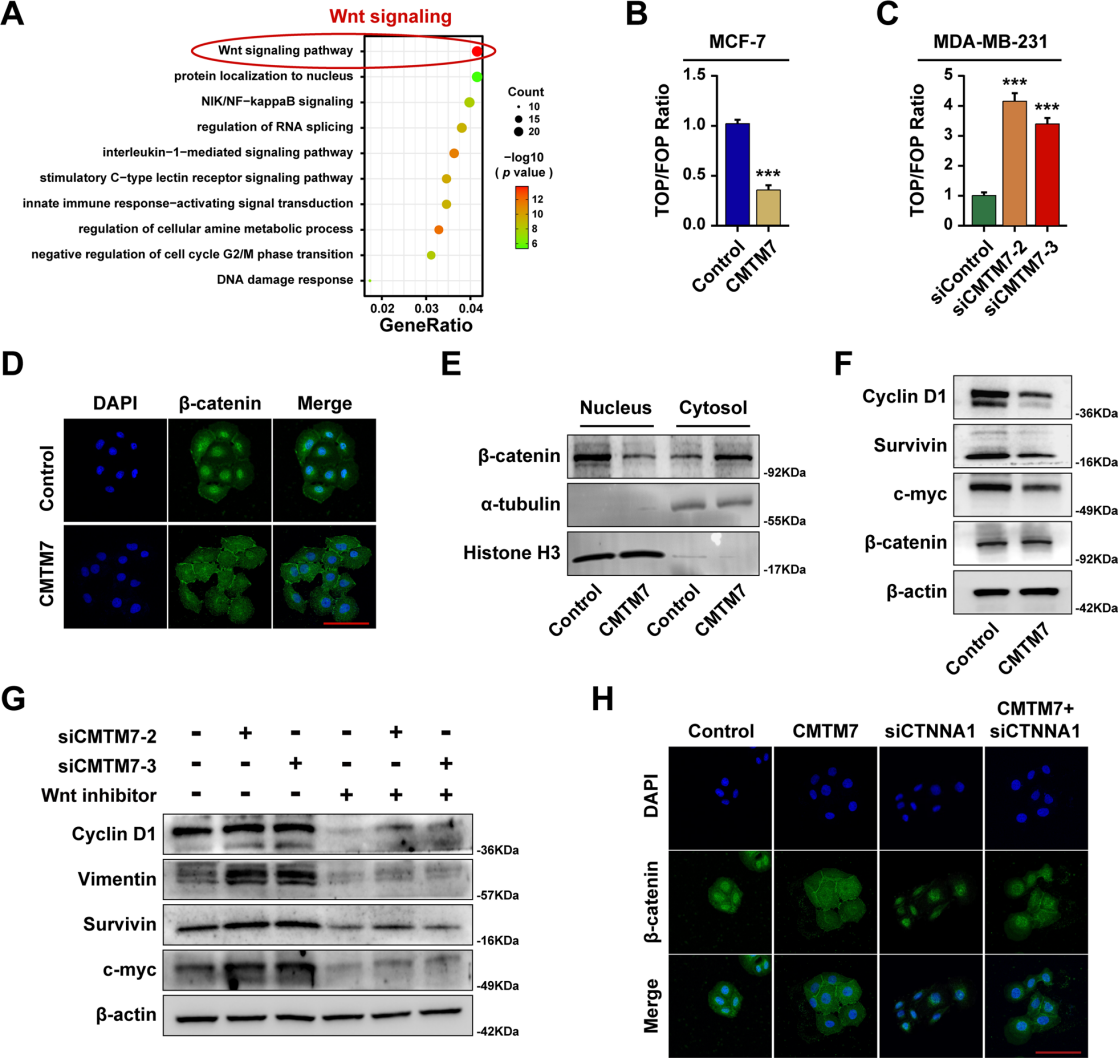
CMTM7 suppressed Wnt signaling by inhibiting the nuclear localization of β-catenin. **A** The GO analysis was performed among the genes identified through mass spectrometry. The TOP/FOP luciferase reporter assay in CMTM7-overexpressed MCF-7 cells (**B**) and in CMTM7-depleted MDA-MB-231 cells (**C**). **D** The localization transformation of β-catenin visualized by IF. **E** The β-catenin expression level in the nucleus and cytoplasm detected by nucleocytoplasmic separation western blot. **F** The expression levels of downstream target genes of Wnt/β-catenin signaling, including cyclin D1, survivin, c-myc, and β-catenin, detected by western blot. **G** The target genes expression after treatment of Wnt/β-catenin signaling inhibitor adavivint in CMTM7-depleted MDA-MB-231 cells. **H** The localization of β-catenin in CMTM7-overexpressed and CTNNA1-depleted MCF-7 cells detected by IF. *p < 0.05; ***p < 0.01

The mass spectrometry results of CMTM7 had addressed plenty of genes that could interact with CMTM7, which was followed by enrichment analysis and focused primarily on Wnt signaling when screened with cancer-related pathways (Fig. 5A). The TOP/FOP luciferase assay was performed to evaluate the regulation of CMTM7 on Wnt signaling, which results showed that CMTM7-overexpression decreased the activity of TOP/FOP luciferase (Fig. 5B), while CMTM7 downregulation increased the activity of TOP/FOP luciferase (Fig. 5C). As extensively studied, Wnt/β-catenin signaling is the classical pathway of Wnt signaling and initiates with the nuclear localization of β-catenin. Thus, immunofluorescence was performed to explore the effect of CMTM7 on the localization of β-catenin, which results showed that nuclear-localized β-catenin was remarkably reduced in CMTM7-overexpressed MCF-7 cells, and instead, β-catenin was abundant on the cell membrane (Fig. 5D). Besides, the nucleo-plasma separation assay showed similar results in CMTM7-overexpressed MCF-7 cells that the β-catenin expression was reduced in nuclear but increased in plasma (Fig. 5E). Furthermore, the expressions of Wnt/β-catenin downstream target genes were detected by western blot, which indicated that CMTM7 downregulated cell cycle-related or EMT-related biomarkers, including Cyclin D1, Survivin, and c-myc but exhibited no effect on the total expression level of β-catenin (Fig. 5F). Then, we further verified the effect of siCMTM7 on Wnt downstream targets with a notably reduction, which could be reversed after the treatment with Wnt signaling inhibitor adavivint (Fig. 5G). The interaction between CMTM7 and CTNNA1 had been demonstrated, and thus, we further investigate the regulation of CTNNA1 on the localization of β-catenin, which result indicated that CMTM7 inhibited the nuclear localization of β-catenin by interacting with CTNNA1 (Fig. 5H). These findings revealed that CMTM7 could inhibit the nuclear localization of β-catenin, thereby inhibiting Wnt/β-catenin signaling.

### CMTM7 was the direct target of miR-182-5p

**Fig. 6:**
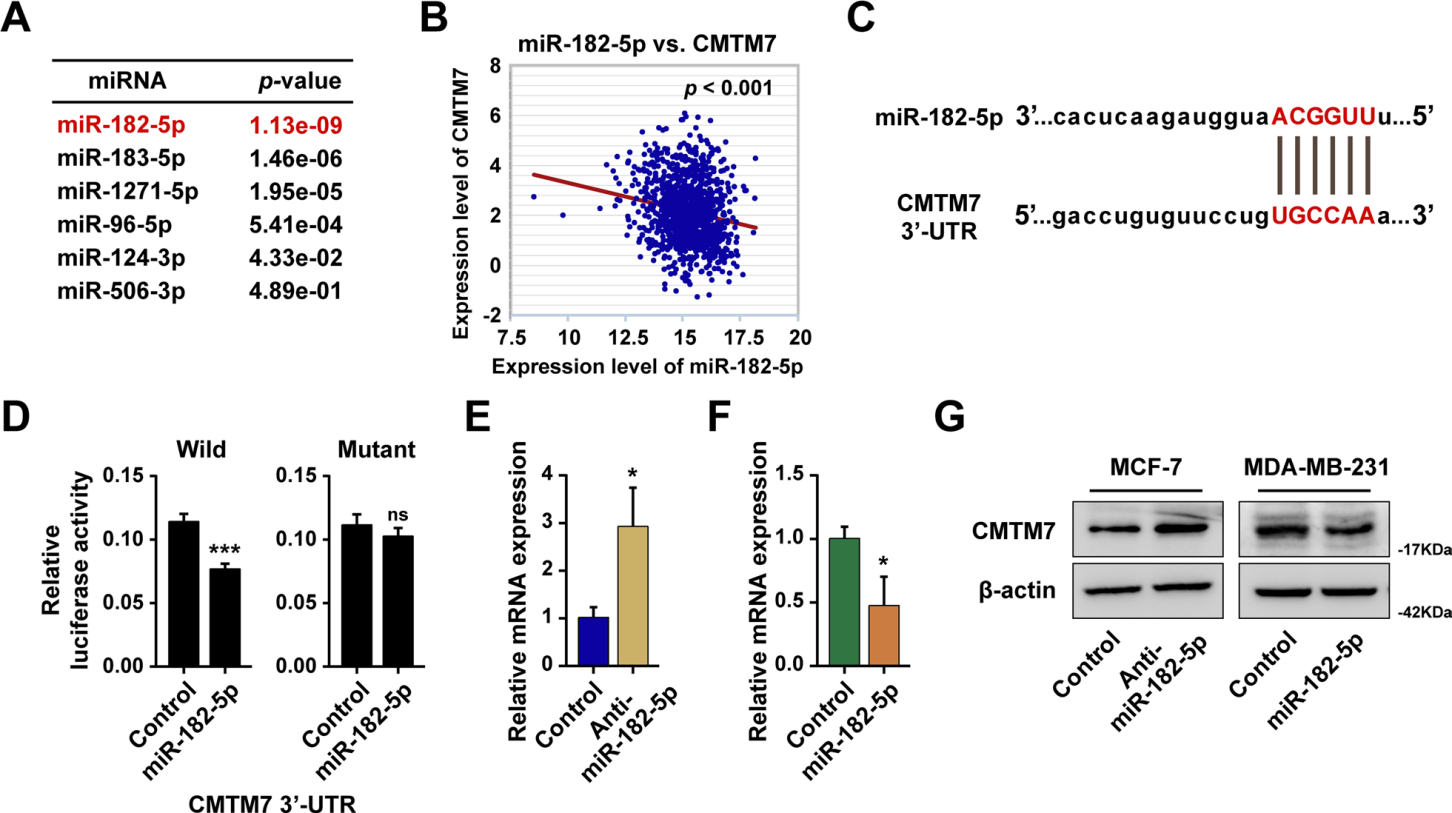
CMTM7 was the direct target of miR-182-5p. **A** The top 6 upstream regulators of CMTM7 predicted by the Starbase website at the rank of the *p* value. **B** The expression correlation between CMTM7 and miR-182-5p. **C** The predicted binding site of miR-182-5p on the 3′UTR region of CMTM7. **D** A dual-luciferase reporter assay was performed to validate the interaction between miR-182-5p and CMTM7. The CMTM7 expression level in miR-182-5p-overexpressed cells (**E**) and miR-182-5p-depleted cells (**F**) detected by RT-qPCR and western blot (**G**). *p < 0.05; ***p < 0.01

To further investigate the upstream regulator of CMTM7, the Starbase website (https://starbase.sysu.edu.cn) was employed to discover the potential miRNAs that could bind to CMTM7 3′UTR region, which results showed that the miR-182-5p was the potential miRNA with the most significant *p* value (Fig. 6A). Besides, miR-182-5p was negatively associated with CMTM7 (Fig. 6B), and a confident banding site had been predicted (Fig. 6C). A luciferase reporter assay confirmed that miR-182-5p could bind to the 3′UTR region of CMTM7 (Fig. 6D). In addition, RT-qPCR and western blot assays illustrated that miR-182-5p could negatively regulate CMTM7 mRNA and protein levels (Fig. 6E–G). Furthermore, miR-182-5p served as a tumor promoter, establishing the opposite performance with CMTM7, and CMTM7-overexpression could reverse the miR-182-5p-induced proliferation and invasion of tumors (Additional file 1: Fig. S7). These findings revealed that miR-182-5p inhibited the expression of CMTM7 by directly targeting its 3′UTR region and functioned as a tumor promoter in breast cancer.

### TCF3 transcriptionally activated miR-182-5p

**Fig. 7:**
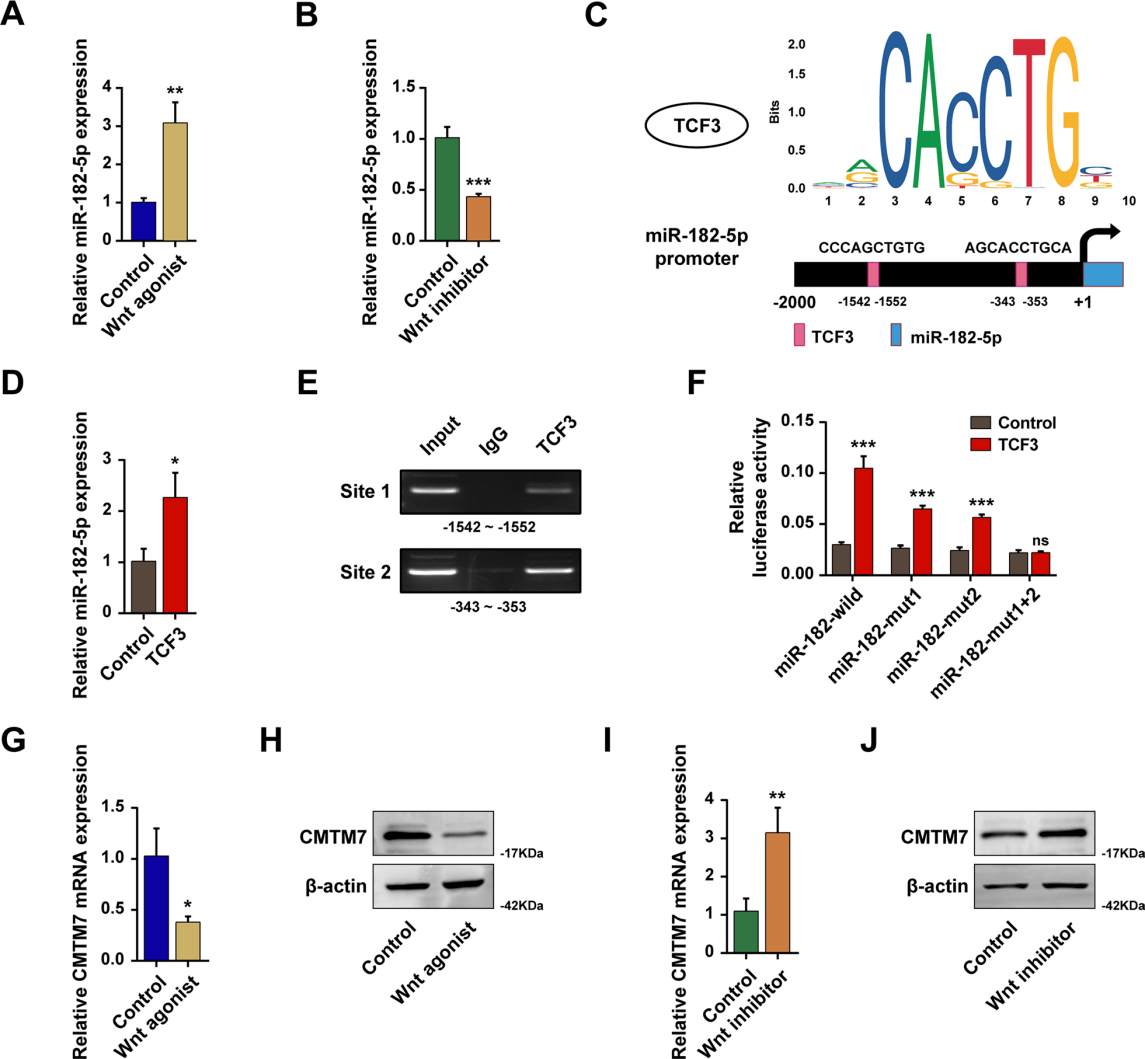
TCF3 transcriptionally activated miR-182-5p. The miR-182-5p expression level after treatment with Wnt agonist 1 (**A**) and Wnt signaling inhibitor adavivint (**B**) detected by RT-qPCR. **C** The predictive binding site of TCF3 on the promoter region of miR-182-5p. **D** The miR-182-5p expression level after transfection of TCF3 expression plasmid. **E** The combination of TCF3 with site1 and site2 by ChIP analysis. **F** A dual-luciferase reporter assay was performed to validate the interaction between TCF3 and miR-182-5p. The CMTM7 expression level after treatment of Wnt agonist detected by RT-qPCR (**G**) and western blot (**H**). The CMTM7 expression level after treatment of Wnt inhibitor detected by RT-qPCR (**I**) and western blot (**J**). *p < 0.05; ***p < 0.01

It has been fully established that TCF3 serves as one of the most functional transcription factors downstream of Wnt/β-catenin signaling, and is able to activate or inhibit many target genes [27]. The miR-182-5p expression level was notably increased after being treated with Wnt signaling agonist 1 (Fig. 7A), while remarkably decreased after being treated with Wnt signaling inhibitor adavivint (Fig. 7B), which indicated that the expression of miR-182-5p was positively correlated with the activation of Wnt signaling. To figure out the regulatory mechanism of Wnt signaling on miR-182-5p, the JASPAR website (https://jaspar.genereg.net/) was employed to predict the potential binding site between TCF3 motif and the promoter of miR-182-5p (Fig. 7C). Besides, it had been observed that the miR-182-5p expression level was significantly upregulated after transfection of TCF3 overexpressed plasmid (Fig. 7D). ChIP analysis verified the regulation of TCF3 on the miR-182-5p promoter and showed TCF3 was mainly enriched in site 2 (-343 to -353) (Fig. 7E). In addition, the luciferase reporter assay confirmed the TCF3 binding on miR-182-5p promoter sites (Fig. 7F). Furthermore, the CMTM7 expression level was significantly decreased after the Wnt agonist treatment (Fig. 7G, H) and was notably increased after the Wnt inhibitor treatment (Fig. 7I, J), which showed the opposite regulatory pattern against miR-182-5p. These results indicated that TCF3 transcriptionally activated miR-182-5p expression by binding to its promoter region.

### CMTM7 was correlated with the prognosis of breast cancer patients

**Fig. 8:**
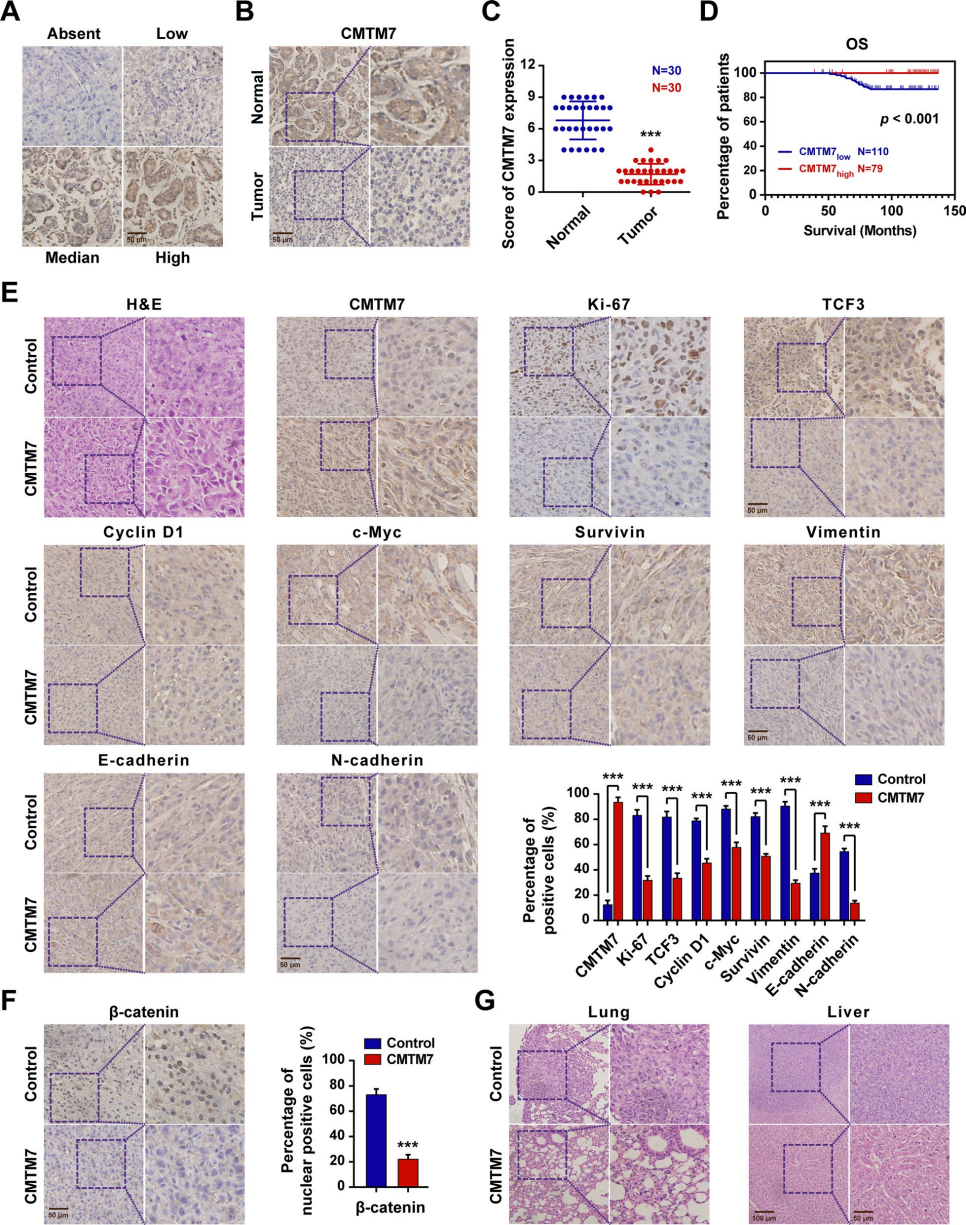
CMTM7 was correlated with the prognosis of breast cancer patients. **A** The standard score of IHC staining. **B** The protein expression level of CMTM7 in breast cancer tissues and paired normal tissues by IHC. **C** The scores of IHC staining in 30 cases of breast cancer patients. **D** The correlation between CMTM7 expression level and OS in 189 cases of patients with breast cancer. **E** H&E staining and CMTM7, Ki-67, TCF3, cyclin D1, c-myc, survivin, vimentin, E-cadherin, and N-cadherin protein expression levels in xenograft. **F** IHC staining of β-catenin in xenograft. **G** H&E staining of lung and liver in xenograft. *p < 0.05; ***p < 0.01

A total number of 30 paired breast cancer and normal tissues were collected for IHC assay to investigate the CMTM7 expression level in breast cancer patients. The standard stain score of IHC is established as Fig. 8A. Consistent with the above results, CMTM7 was remarkably downregulated in breast cancer tissues compared to normal tissues (Fig. 8B, C). Besides, survival analysis on the clinical profiles of 189 patients illustrated that patients with higher CMTM7 expression were correlated with better clinical outcomes (Fig. 8D). Furthermore, the tumors obtained from SCID mice were sectioned and underwent IHC stain to verify the expression levels of related genes in vivo, which results showed that the Ki-67, TCF3, cyclin D1, c-Myc, survivin, vimentin, and N-cadherin expression levels were significantly downregulated in mice with CMTM7-overexpression, while E-cadherin expression was upregulated (Fig. 8E). In particular, the nuclear-localized β-catenin was decreased (Fig. 8F). H&E staining also indicated that the mice of control group exhibited obvious lung and liver metastasis, which were almost invisible in CMTM7-overexpressed group (Fig. 8G). These results revealed that CMTM7 was correlated with better breast cancer prognosis, and all these findings had been further validated through the experiments of xenograft.

## Discussion

**Fig. 9:**
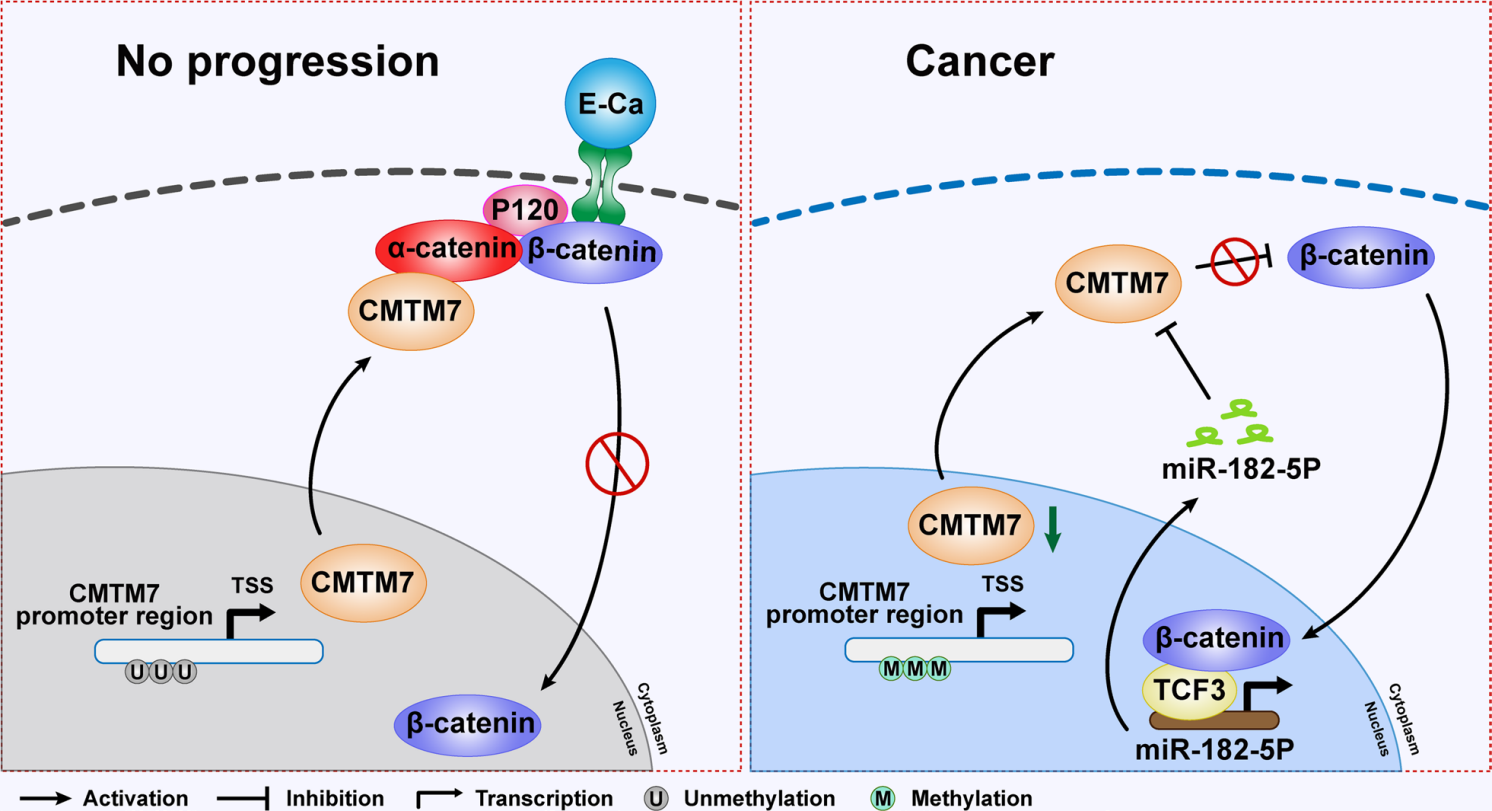
A model of the role of CMTM7 in breast cancer

In the current study, we revealed a feedback loop formed by miR-182-5p/CMTM7/CTNNA1/CTNNB1/TCF3 in breast cancer progression. Normally, CMTM7 is hypomethylated and interacts with CTNNA1, which is able to form a CTNNA1/CTNNB1 complex at the cell membrane to recruit E-cadherin, and thus reduces the nuclear localization of CTNNB1, and inhibits Wnt/β-catenin signaling. However, in breast cancer, CMTM7 is hypermethylated and loses the inhibition of Wnt/β-catenin signaling. Besides, the Wnt/β-catenin signaling downstream transcriptional factor TCF3 can transcriptionally activate miR-182-5p, which serves as the upstream regulator of CMTM7 by binding with its 3′UTR region (Fig. 9). The existence of the feedback loop uncovered the mechanism of CMTM7 in breast cancer and may provide promising strategies for breast cancer therapy.

CMTM7 was found to be significantly downregulated in both breast cancer tissues and cell lines compared with normal breast cancer tissues and cell lines in our study; however, it is interesting that CMTM7 expressed differentially among breast cancer subtypes. As shown in Fig. 1I, J, comparing with other types, the expression of CMTM7 seemed to be higher in basal-like breast cancer type, which is a more aggressive subtype, and might be abnormal for a tumor suppressor should be performed. However, basal-like breast cells are characterized by the basal gene expression and with mesenchymal characteristics [28], the expression pattern of CMTM7 in basal-like breast cells is consistent with its nature of mesenchymal regulatory factors, which needs further investigation to figure out. Besides, the same expression pattern has been reported in a previous study [29].

Increasingly evidence has revealed the crucial roles of epigenetic regulation in gene expression. Jiao et al. illustrated the SEPT9 promoter methylation and its role in regulating radio resistance in cervical cancer [30]. Tan et al. reported that the promoter methylation of tumor suppressor DRD2 was involved in NF-κB signaling in breast cancer [31]. Interestingly, Li et al. demonstrated that the CMTM7 downregulation was associated with the methylation of the CpG island on its promoter region and verified in multiple cancer types [13]. In our study, we investigated the methylation of CMTM7 CpG island through BSP sequencing among breast cancer cells and tissues. The results indicated that CMTM7 was hypermethylated in breast cancer, leading to the CMTM7 downregulation.

AlphaE-catenin (CTNNA1) is a member of Alpha-catenins that serve as mechanosensing proteins, which can modify the combination between the cadherin and the actin cytoskeleton [32]. CTNNA1 has been fully established as a tumor suppressor that inhibits proliferation and invasion of various cancers [33]. Furthermore, CTNNA1 is able to form an E-cadherin/catenin/cytoskeleton complex with CTNNB1 (β-catenin) and thus involves in the regulation of EMT progression [34]. In current study, CTNNA1 was found to interact with CMTM7 and inhibited the nuclear localization of β-catenin.

microRNAs (miRNAs) are a class of noncoding RNAs that are deeply involved in fundamental biological processes [35]. The dysregulation of miRNAs on their target genes in cancer progression has become the research hotspot in recent years [36]. The miR-29b-3p inhibited thyroid cancer invasion and migration by regulating COL1A1 and COL5A1 [37]. The miR-520a-5p plays an essential role in regulating gemcitabine resistance by directly targeting PPP5C in pancreatic cancer [38]. Furthermore, a previous study demonstrated the miR-182-5p regulation on CMTM7 in breast cancer, demonstrating that EVs-miR-182-5p promoted tumor progression by regulating the CMTM7/EGFR/AKT signaling axis [39]. We obtained consistent results, but there was a significant difference. Our study revealed for the first time that CMTM7 regulates the nuclear localization of β-catenin and is involved in Wnt signaling. Besides, the transcriptional regulation of TCF3 on miR-182-5p was first reported. These findings indicated the existence of the miR-182-5p/CMTM7/TCF3 feedback loop and were of great significance.

TCF3 belongs to the E-protein family, which involves in diverse developmental processes [40]. TCF3 performs essential roles in cancer development as the main target gene of Wnt signaling. Gui T et al. reported that TCF3 is silenced by DNMT3B and EZH2 epigenetically and inhibits tumor progression in endometrial cancer [41]. Besides, TCF3 can also be transcriptionally active WDR5 and involved in miR-17-5p and HOXA11-AS activation, which results in poorer prognosis in gastric cancer [42]. Moreover, Jia et al. demonstrated that TCF3-activated FAM201A promoted aggressive phenotypes of triple-negative breast cancer cells by regulating TNKS1BP1 expression [27]. In the current study, TCF3 could transcriptionally activate miR-182-5p by binding with its promoter region, verified by luciferase reporter assay and ChIP analysis.

## Conclusion

In our study, we discovered that CMTM7 is a tumor suppressor that is regulated by methylation of its CpG island. Besides, CMTM7 served as the target of miR-182-5p and interacted with CTNNA1, leading to the decreased nuclear localization of β-catenin, thus involved in inhibiting Wnt/β-catenin signaling. Finally, the downstream transcriptional factor of Wnt/β-catenin signaling, TCF3, could transcriptionally activate miR-182-5p. In summary, our study uncovered the existence of the miR-182-5p/CMTM7/CTNNA1/CTNNB1/TCF3 regulation loop in breast cancer, which may provide novel targets and promising strategies for breast cancer therapy.

## Supplementary Information

**Additional file 1.** Supplementary tables and figures.

**Additional file 2.** The dynamic migration video of control group in MCF-7 cells.

**Additional file 3.** The dynamic migration video of CMTM7-overexpressed MCF-7 cells.

**Additional file 4.** The dynamic migration video of control group in MDA-MB-231 cells.

**Additional file 5.** The dynamic migration video of MDA-MB-231 cells transfected with siCMTM7-2.

**Additional file 6.** The dynamic migration video of MDA-MB-231 cells transfected with siCMTM7-3.

## Data Availability

All data generated or analyzed during this study are included in this article and its supplementary information files.
