# Supplementary material for: CMTM7 inhibits breast cancer progression by regulating Wnt/β-catenin signaling
Source: Breast Cancer Res. 2023 Feb 24;25:22. doi: 10.1186/s13058-023-01620-9 (PMC9960403; doi:10.1186/s13058-023-01620-9)

**Table S1. Oligonucleotides of miRNAs and siRNAs.**

| Mimic control | UGUACCAAUUUCCAGUGGAGAU |
| --- | --- |
| Inhibitor control | AUGGUGUUAUCAAGUGUAACAG |
| miR-182-5p mimics | UUUGGCAAUGGUAGAACUCACACU |
| miR-182-5p inhibitor | AGUGUGAGUUCUACCAUUGCCAAA |
| siControl | UUCUCCGAACGUGUCACGU |
| siCMTM7-1 | GCATATGGCTGTCCTATAA |
| siCMTM7-2 | GCGACTTGATAATGATCCT |
| siCMTM7-3 | GCGCCTACAGCTACTTTGA |
| siCTNNA1 | AGAGAGGTCGTTCTAAGAA |

**Table S2. Oligonucleotides used for RT-qPCR.**

| **Name** | **Sequence (5’ to 3’)** |
| --- | --- |
| CMTM7 up  CMTM7 low | TGCGACTTGATAATGATCCTCG  CACGCGGTAGAAGCGGAAG |
| GAPDH up  GAPDH low | CAAGGTCATCCATGACAACTTTG  GTCCACCACCCTGTTGCTGTAG |

**Table S3. Oligonucleotides used for ChIP and methylation-specific PCR.**

| **Name** | **Sequence (5’ to 3’)** |
| --- | --- |
| miR-182 site 1 up  miR-182 site 1 low | CCATTATCCCAGCCTCTGCC  CCTGCTTTGGGACATCAGCA |
| miR-182 site 2 up  miR-182 site 2 low | AAATGGAGCAGTGACGTGGG  TGTAACCTGCAGTTCCTGTGG |
| BSP up  BSP low | GGACTGCCGCTACCTTTCTGGA  TTGGAGGGGCTGCTGGAC |


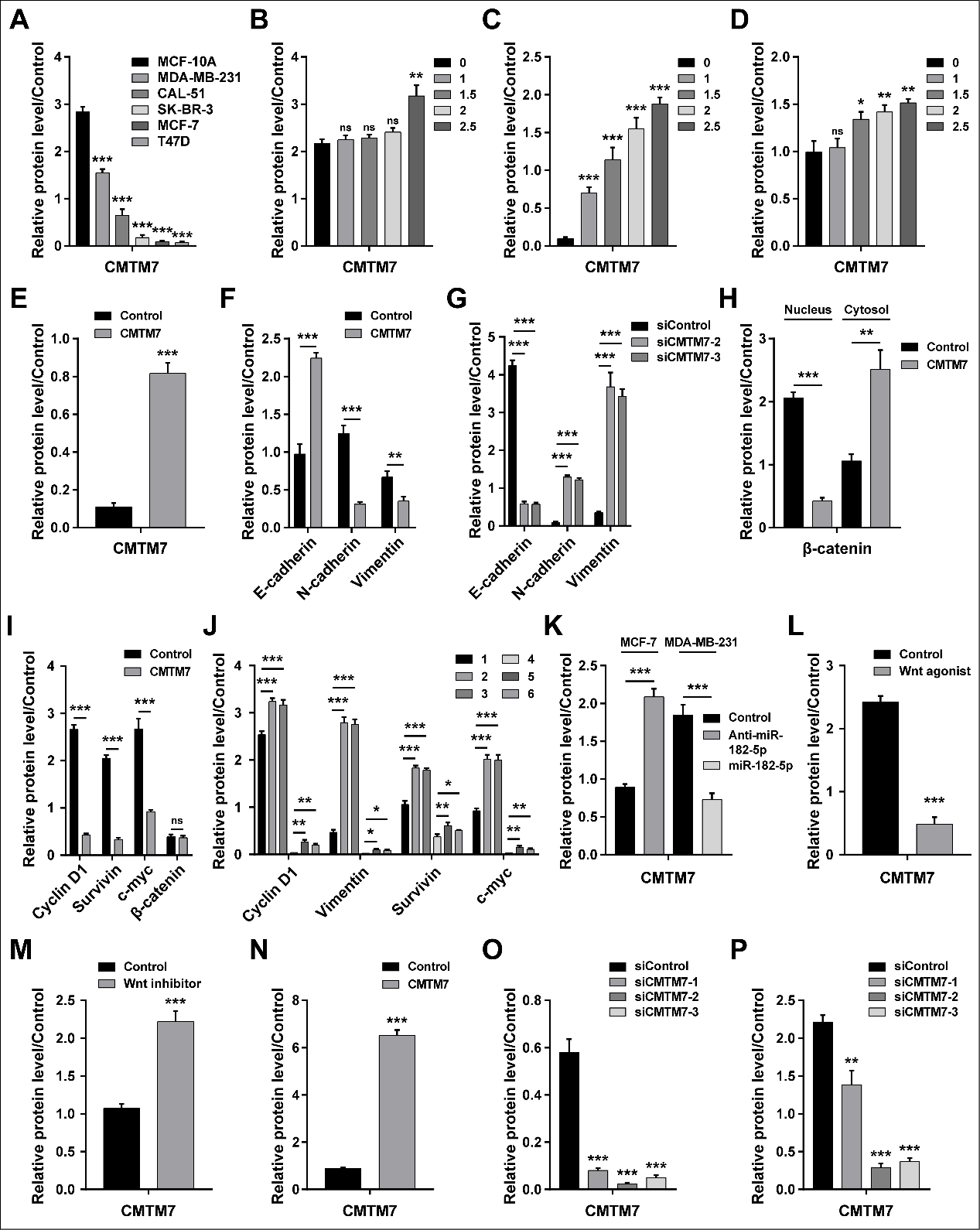


**Fig. S1. The statistical analysis of immunoblots.**

The scale values of each single stripe were measured by Image J software and normalized by β-actin, including (A) Figure 1J, (B) Figure 2C, (C) Figure 2D, (D) Figure 2E, (E) Figure 3A, (F) Figure 3G (left), (G) Figure 3G (right), (H) Figure 5E, (I) Figure 5F, (J) Figure 5G, (K) Figure 6G, (L) Figure 7H, (M) Figure 7J, (N) Figure S3A, (O) Figure S4A and (P) Figure S5A. *p < 0.05, ***p < 0.01.


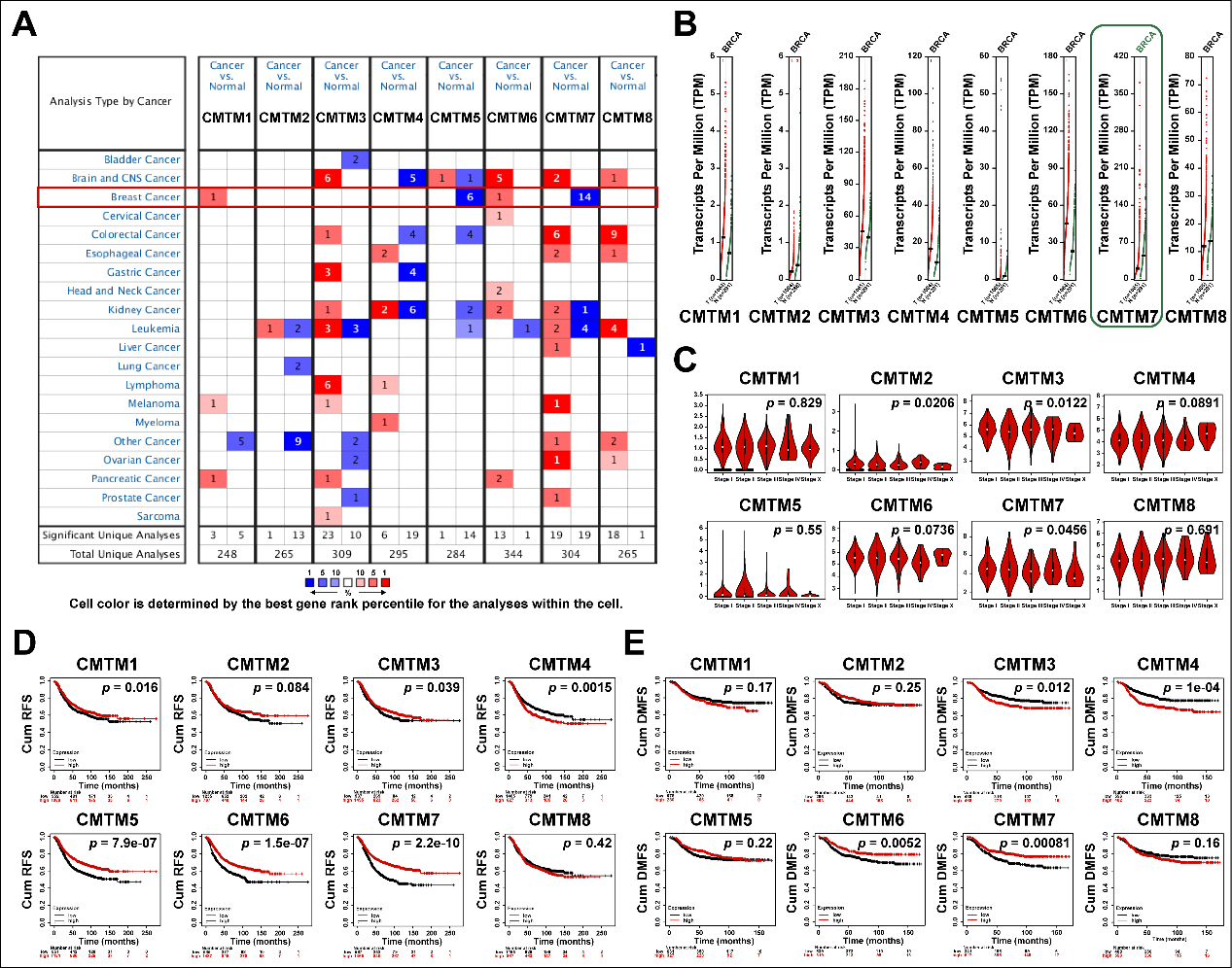


**Fig. S2. The expression pattern of CMTM family in cancer.**

(A) CMTM expression levels in multiple cancer types analyzed by Oncomine. (B) CMTM1-8 expression levels in breast cancer visualized by GEPIA. (C) CMTM1-8 expression pattern in different breast cancer stages. The correlation between CMTMs and prognosis analyzed by KM plotter, including RFS (D) and DMFS (E).


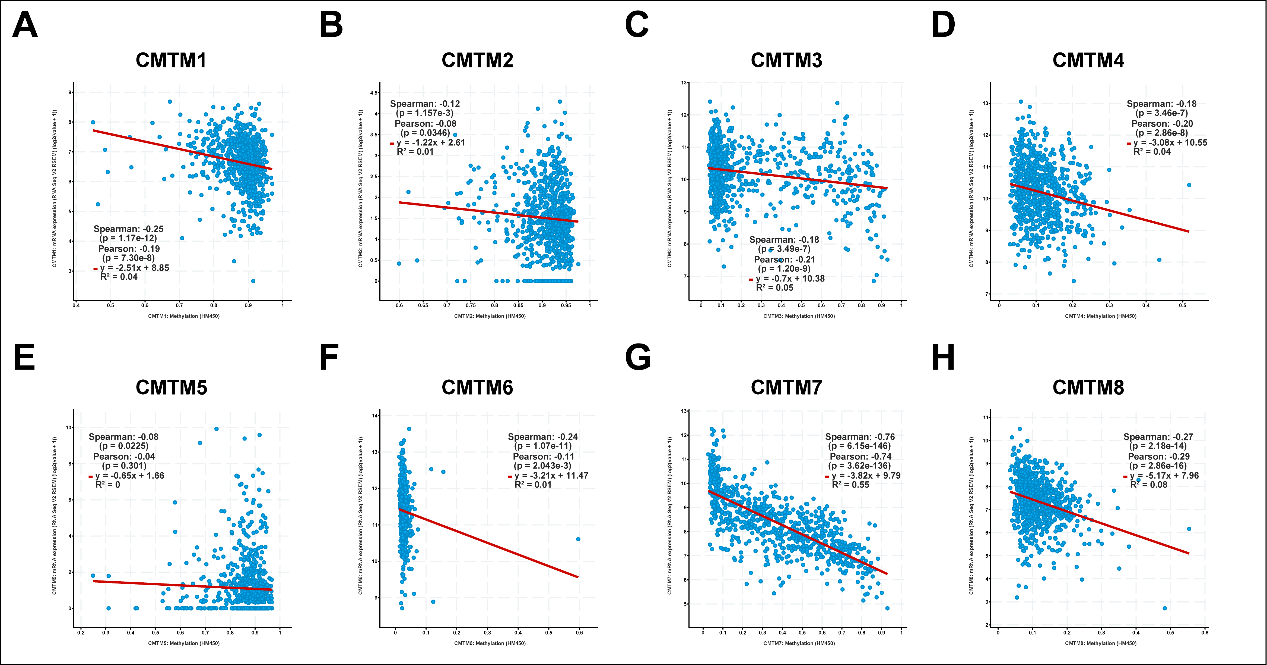


**Fig. S3. The correlation between DNA methylation and gene expression.**

The correlation between DNA methylation and the expression level of CMTM1 (A), CMTM2 (B), CMTM3 (C), CMTM4 (D), CMTM5 (E), CMTM6 (F), CMTM7 (G), and CMTM8 (H).


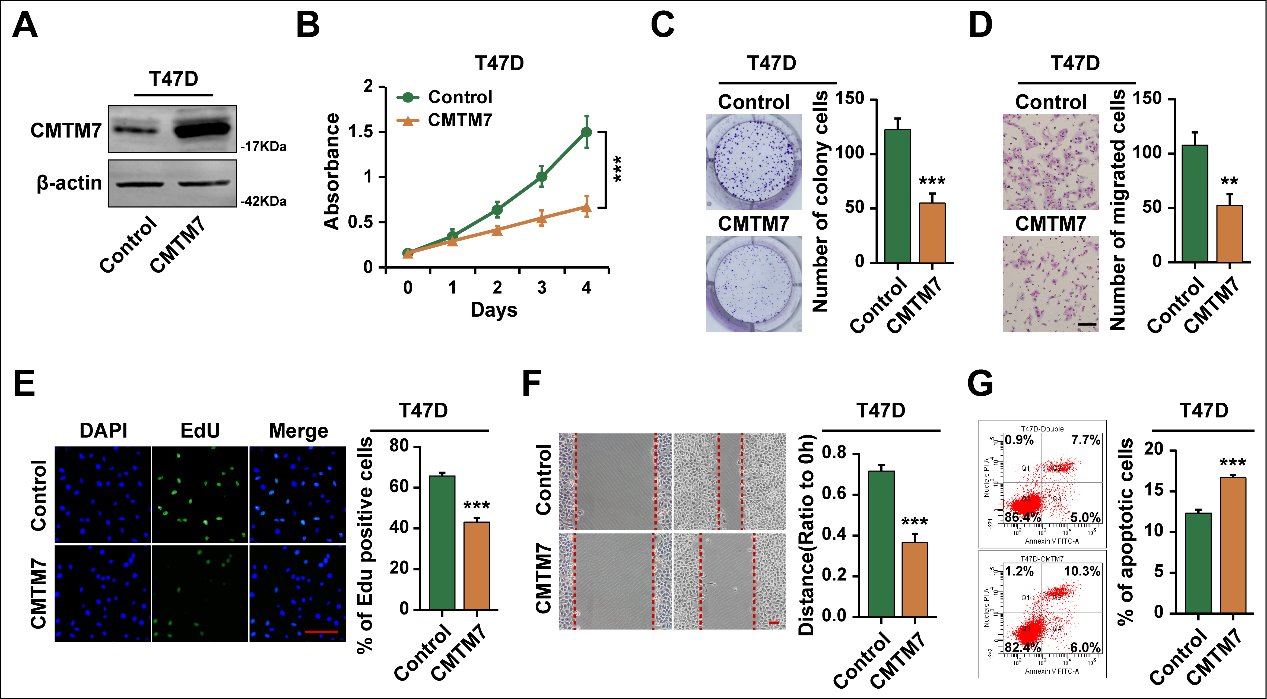


**Fig. S4. CMTM7 inhibited the cell proliferation and invasion of T47D cells.**

(A) The construction of CMTM7-overexpressed T47D cells verified by western blot. The cell proliferation assays were performed, including MTT (B), colony formation (C) and EdU assay (E). The cell invasion and migration assays were performed, including transwell assay (D) and scratch assay (F). (G) The apoptotic cells were analyzed by flow cytometry. *p < 0.05, ***p < 0.01.


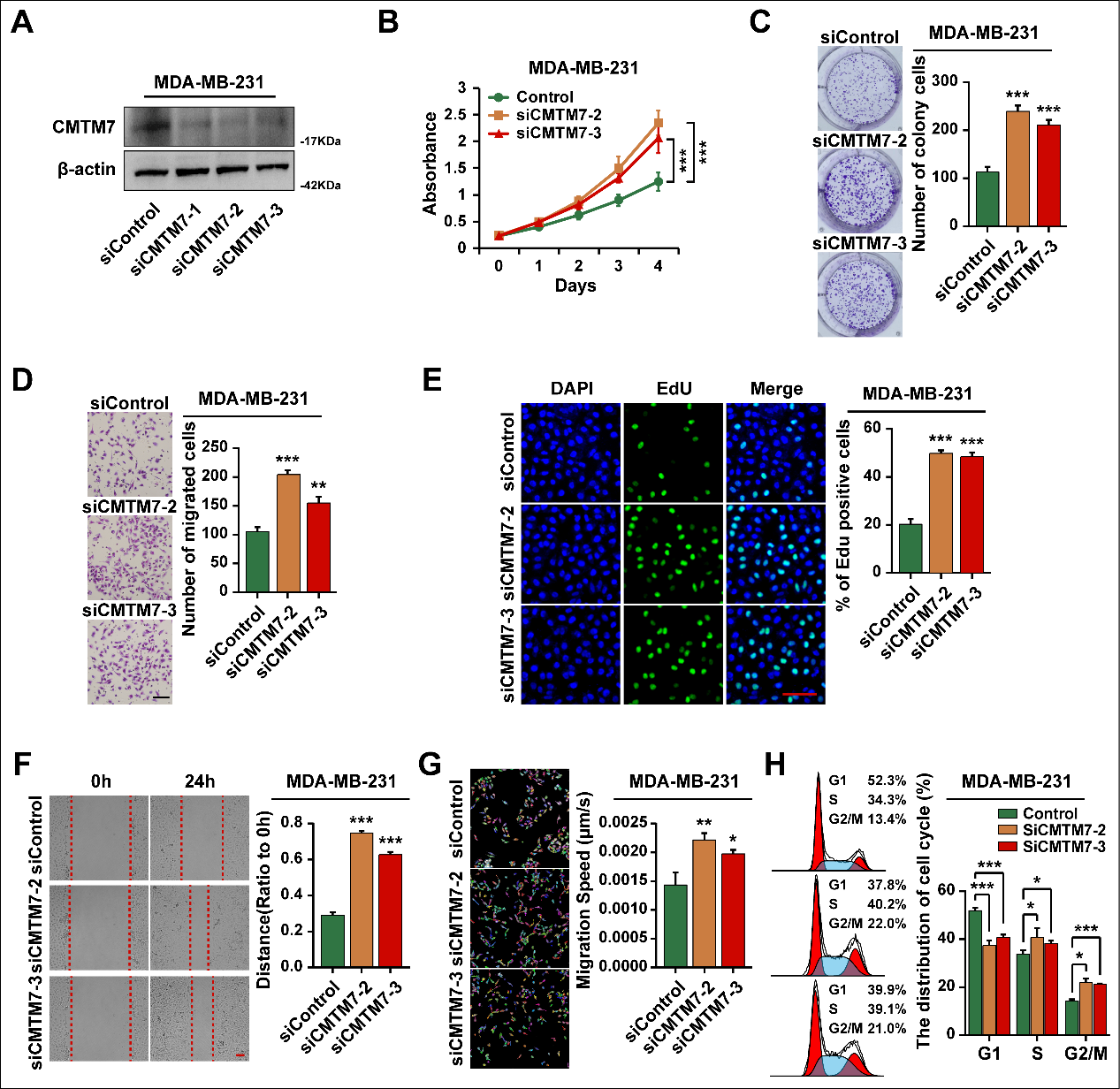


**Fig. S5. CMTM7-deleption increased the proliferation and invasion of MDA-MB-231 cells.**

(A) The siRNAs were utilized to generate CMTM7-delepted MDA-MB-231 cells, and the downregulation of CMTM7 was confirmed by western blot. The cell proliferation assays were performed, including MTT (B), colony formation (C) and EdU assay (E). The cell invasion and migration assays were performed, including transwell assay (D), scratch assay (F) and single cell time-lapse imaging assay (G, Video 3-5). (H) The distribution of cell cycle was detected by flow cytometry. *p < 0.05, ***p < 0.01.


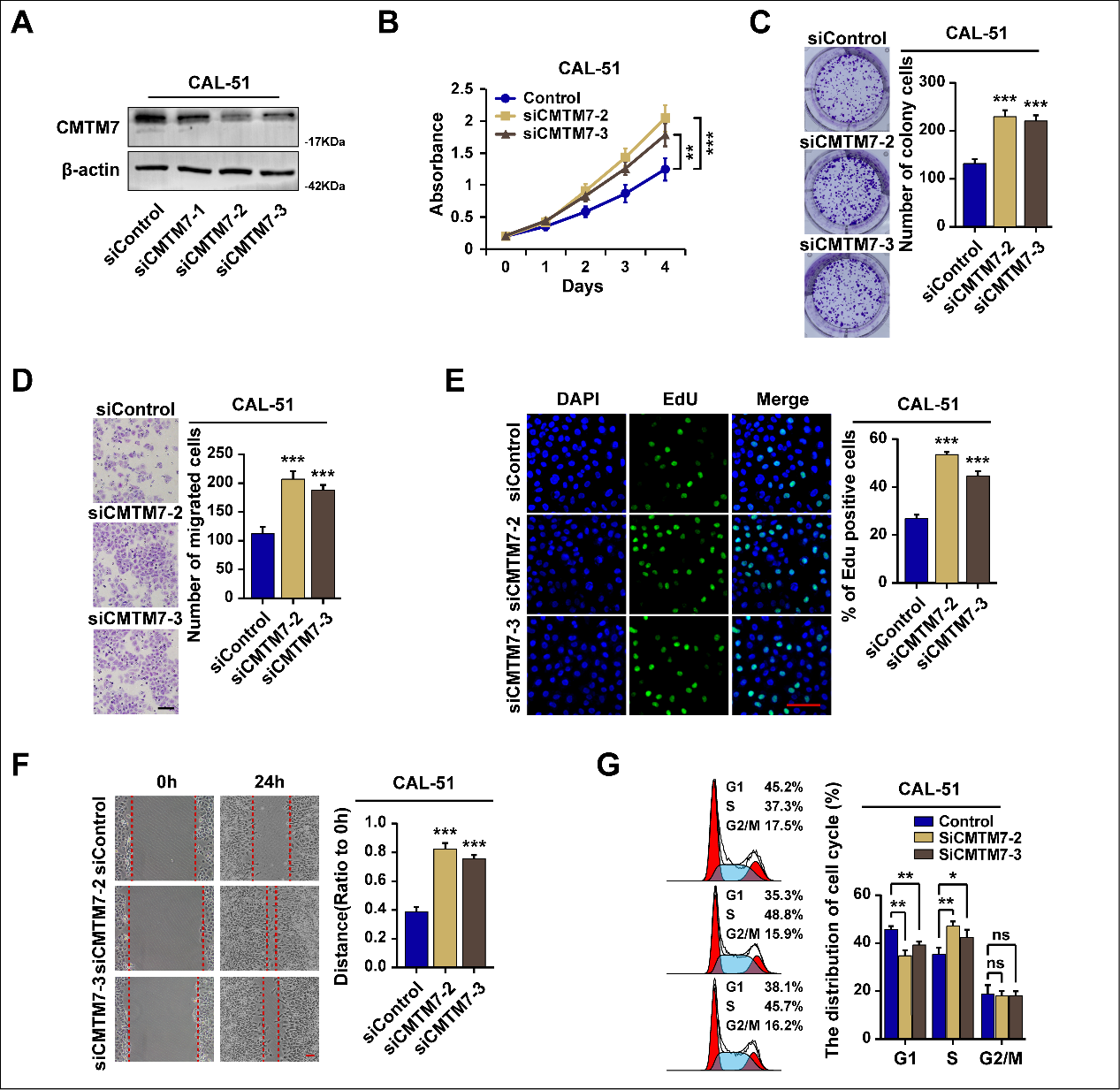


**Fig. S6. CMTM7-deleption increased the proliferation and invasion of CAL-51 cells.**

(A) The siRNAs were utilized to generate CMTM7-delepted CAL-51 cells, and the downregulation of CMTM7 was confirmed by western blot. The cell proliferation assays were performed, including MTT (B), colony formation (C), and EdU assay (E). The cell invasion and migration assays were performed, including transwell assay (D) and scratch assay (F). (G) The distribution of cell cycle was detected by flow cytometry. *p < 0.05, ***p < 0.01.


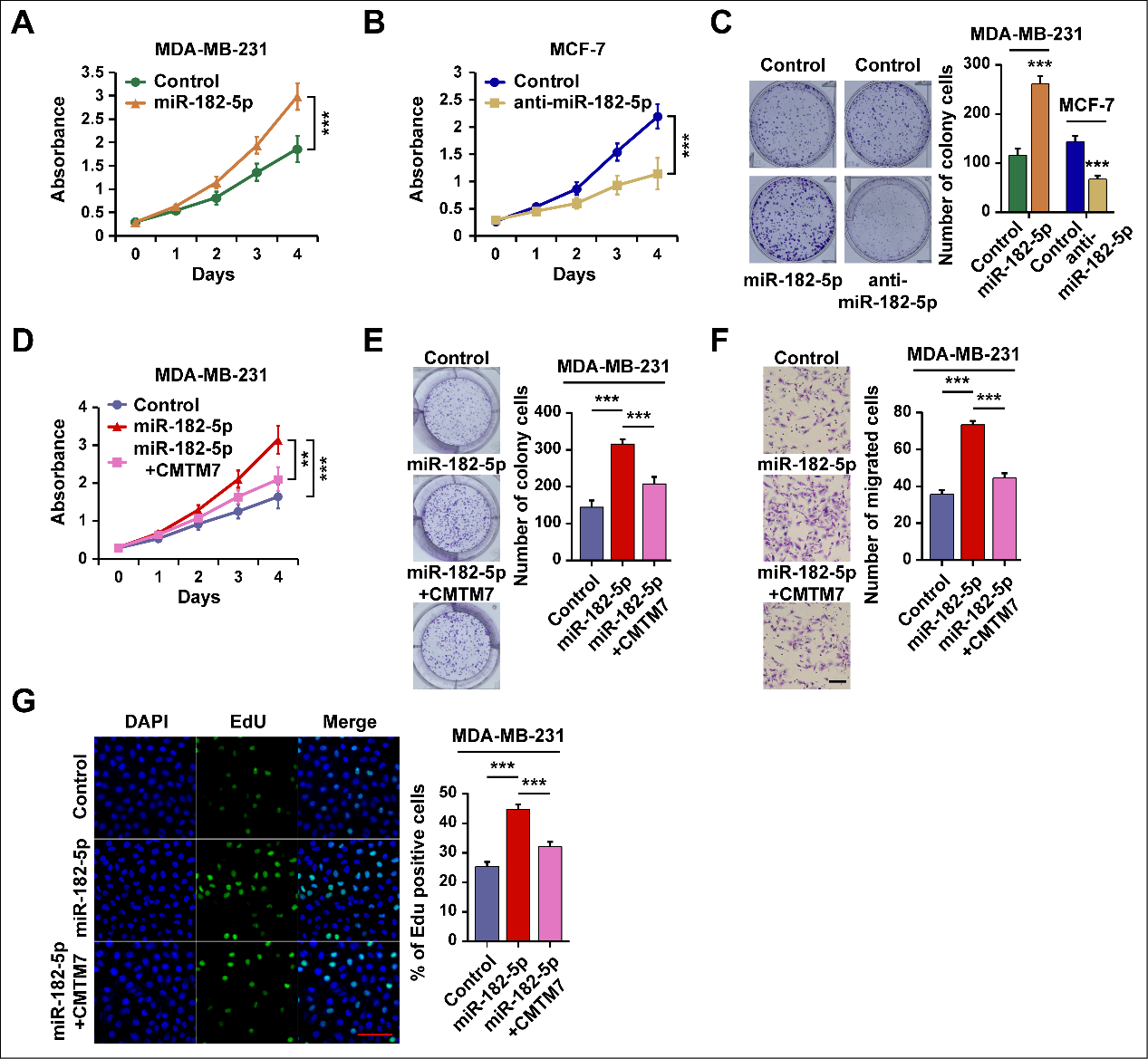


**Fig. S7. miR-182-5p promoted cancer progression by targeting CMTM7.**

MTT assay performed in miR-182-5p-overexpressed MCF-7 cells (A) and in miR-182-5p-delepted MDA-MB-231 cells (B). (C) The colony formation assay performed in miR-182-5p-overexpressed and miR-182-5p-delepted cells. The proliferation assays performed in miR-182-5p-overexpressed cells after transfection of CMTM7 detected by MTT (D), colony formation (E), and EdU (G). (F) The transwell assay performed in miR-182-5p-overexpressed cells after transfection of CMTM7. *p < 0.05, ***p < 0.01.

**Video 1. The dynamic migration video of Control group in MCF-7 cells.**

**Video 2. The dynamic migration video of CMTM7-overexpressed MCF-7 cells.**

**Video 3. The dynamic migration video of Control group in MDA-MB-231 cells.**

**Video 4. The dynamic migration video of MDA-MB-231 cells transfected with siCMTM7-2.**

**Video 5. The dynamic migration video of MDA-MB-231 cells transfected with siCMTM7-3.**

**Original blot images**

Figure 1K


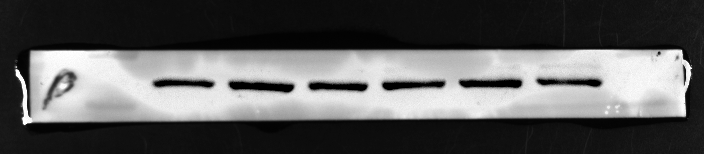

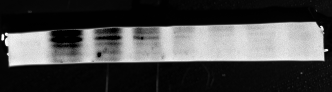


Figure 2C


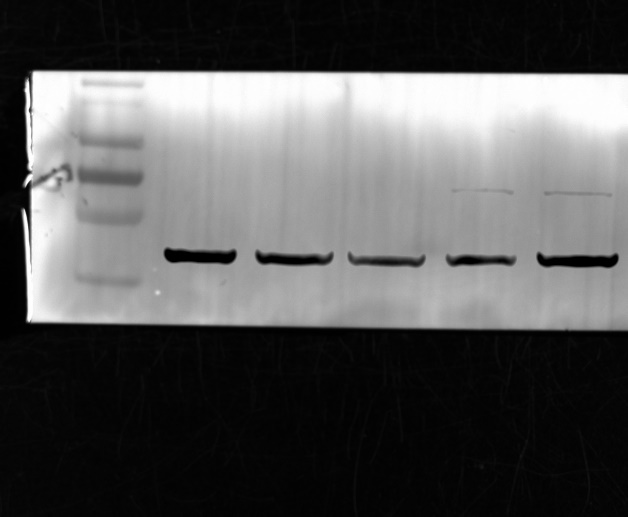

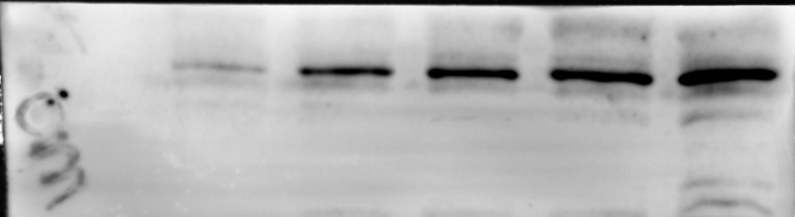

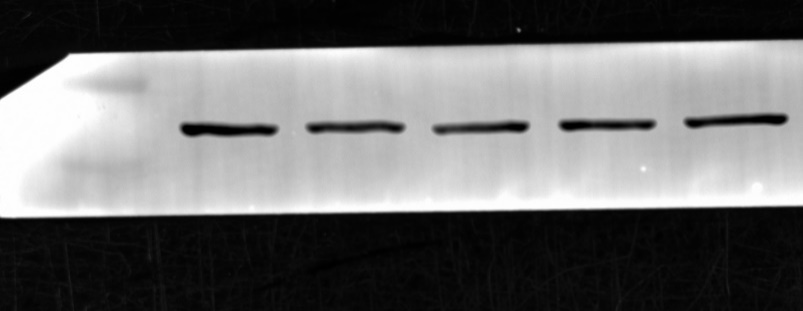

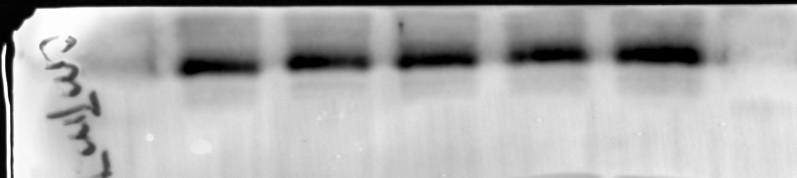
Figure 2D

Figure 2E


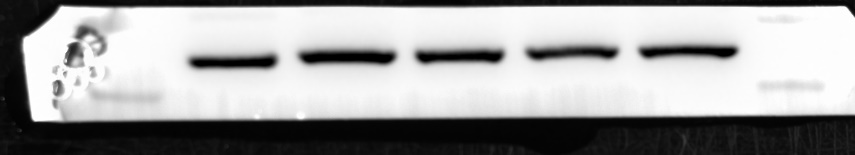

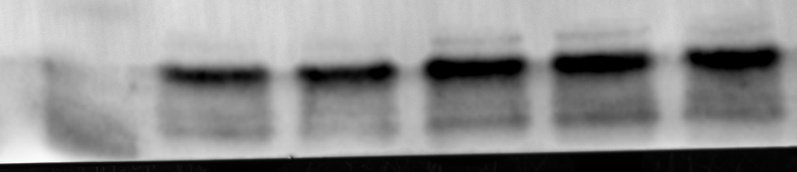


Figure 3A


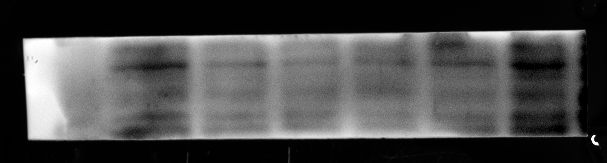


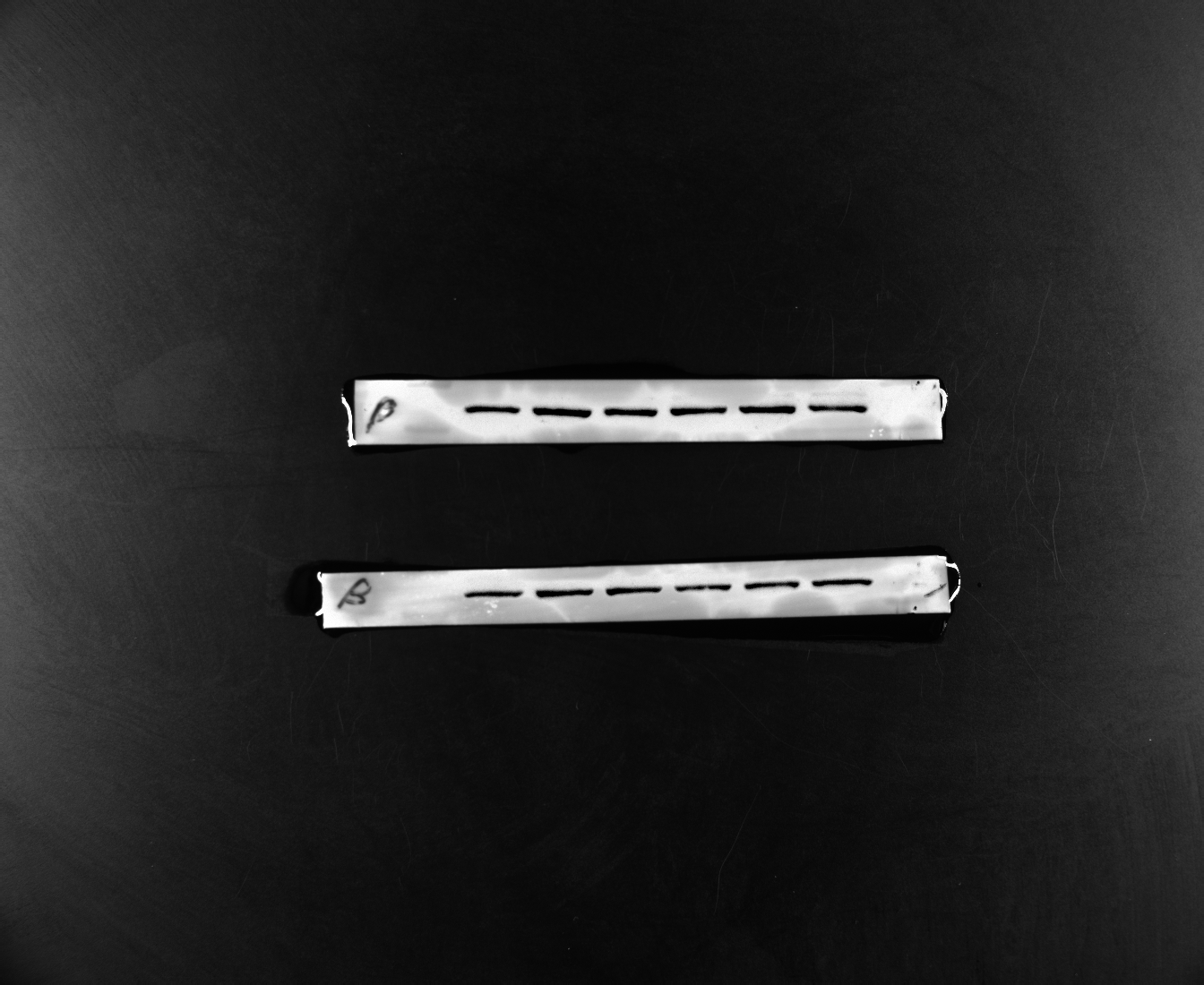


Figure 3G (left)


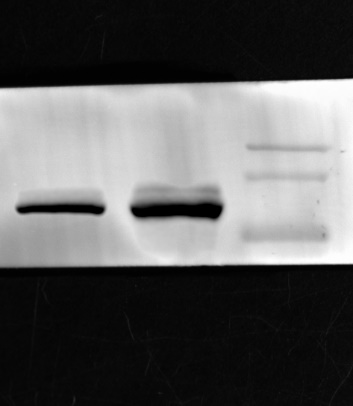


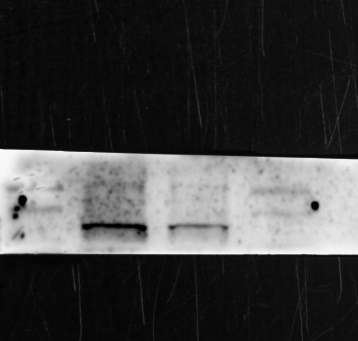


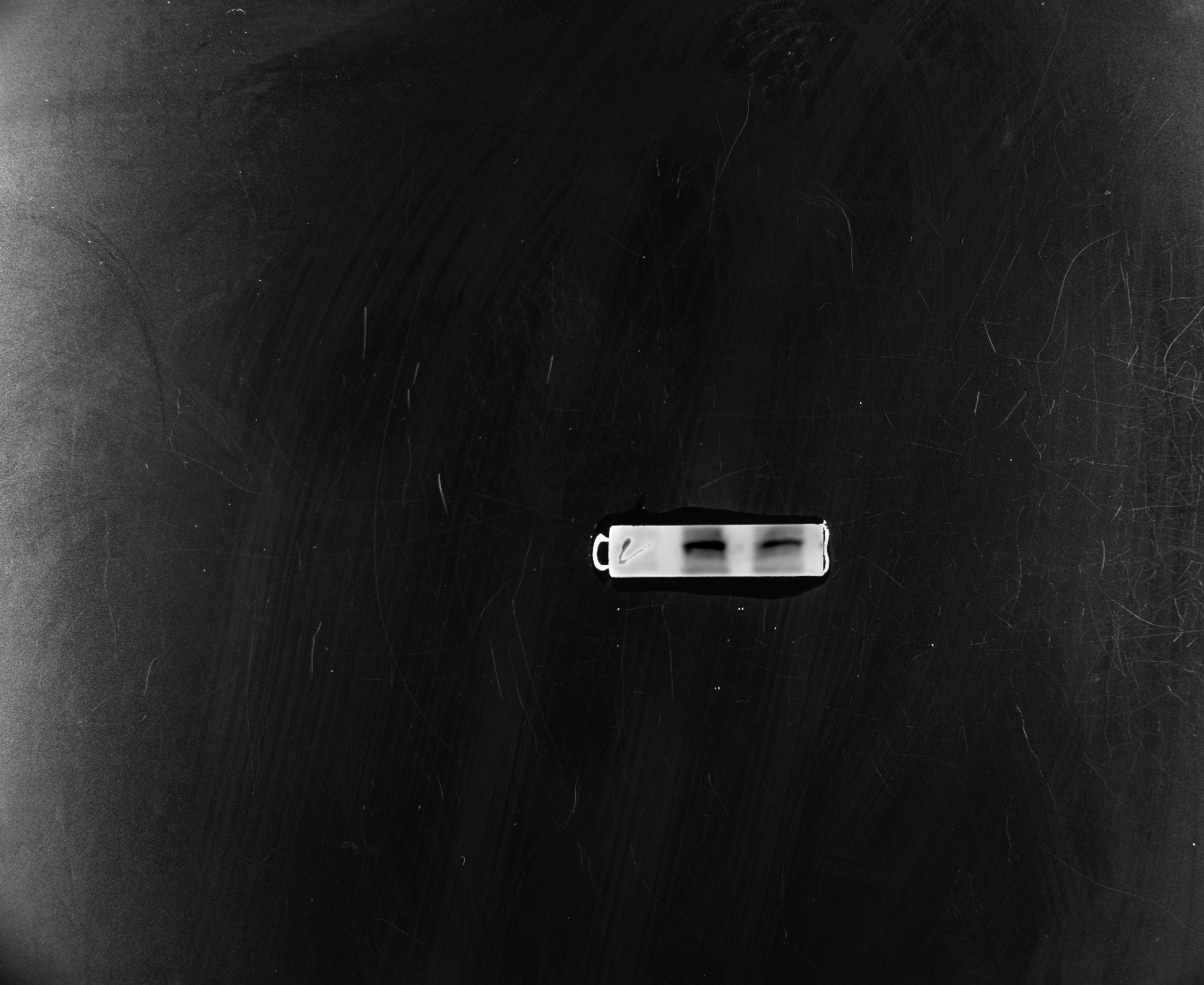


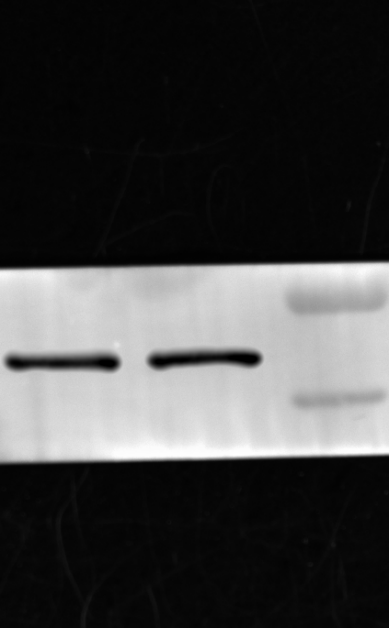


Figure 3G (right)


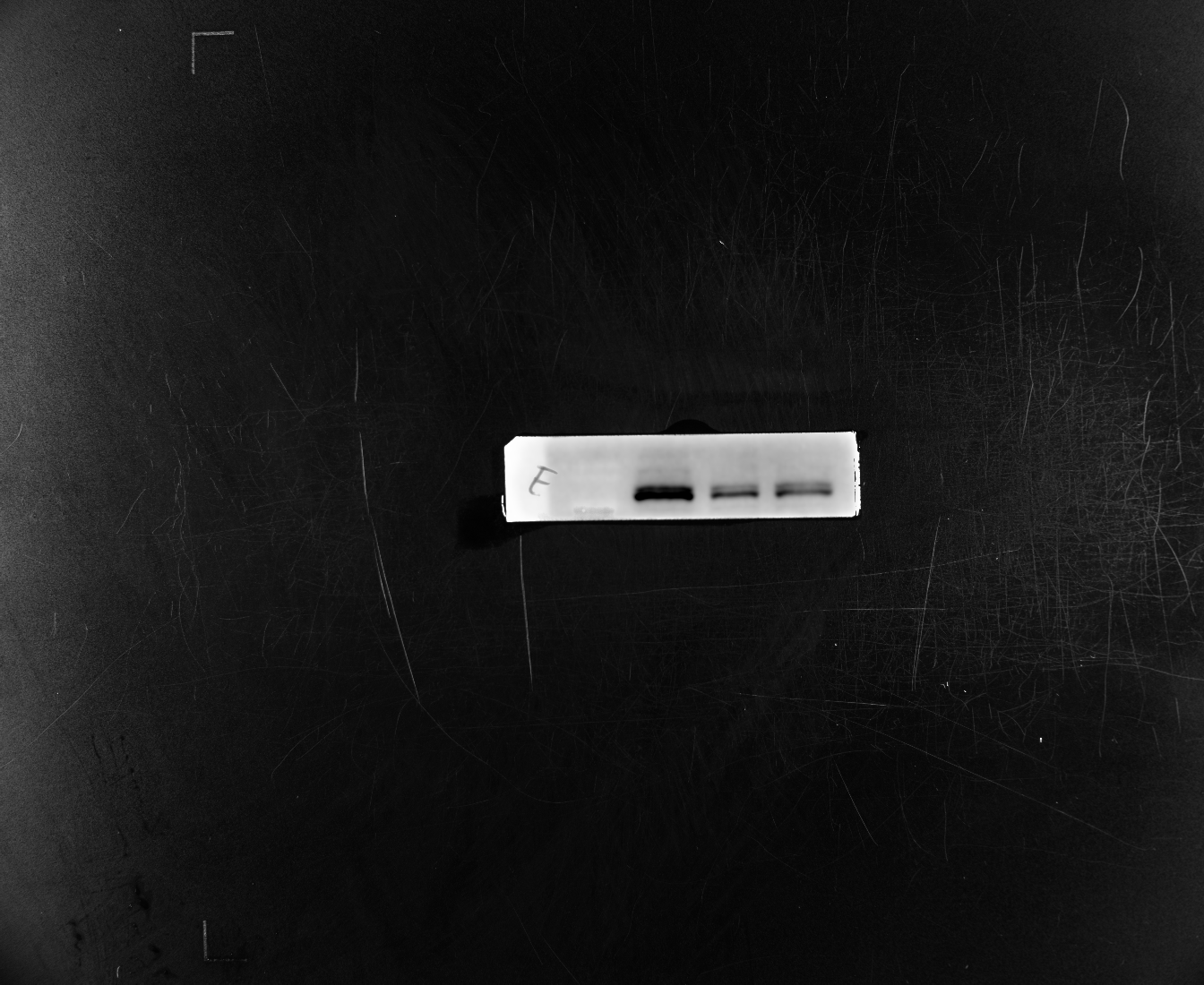


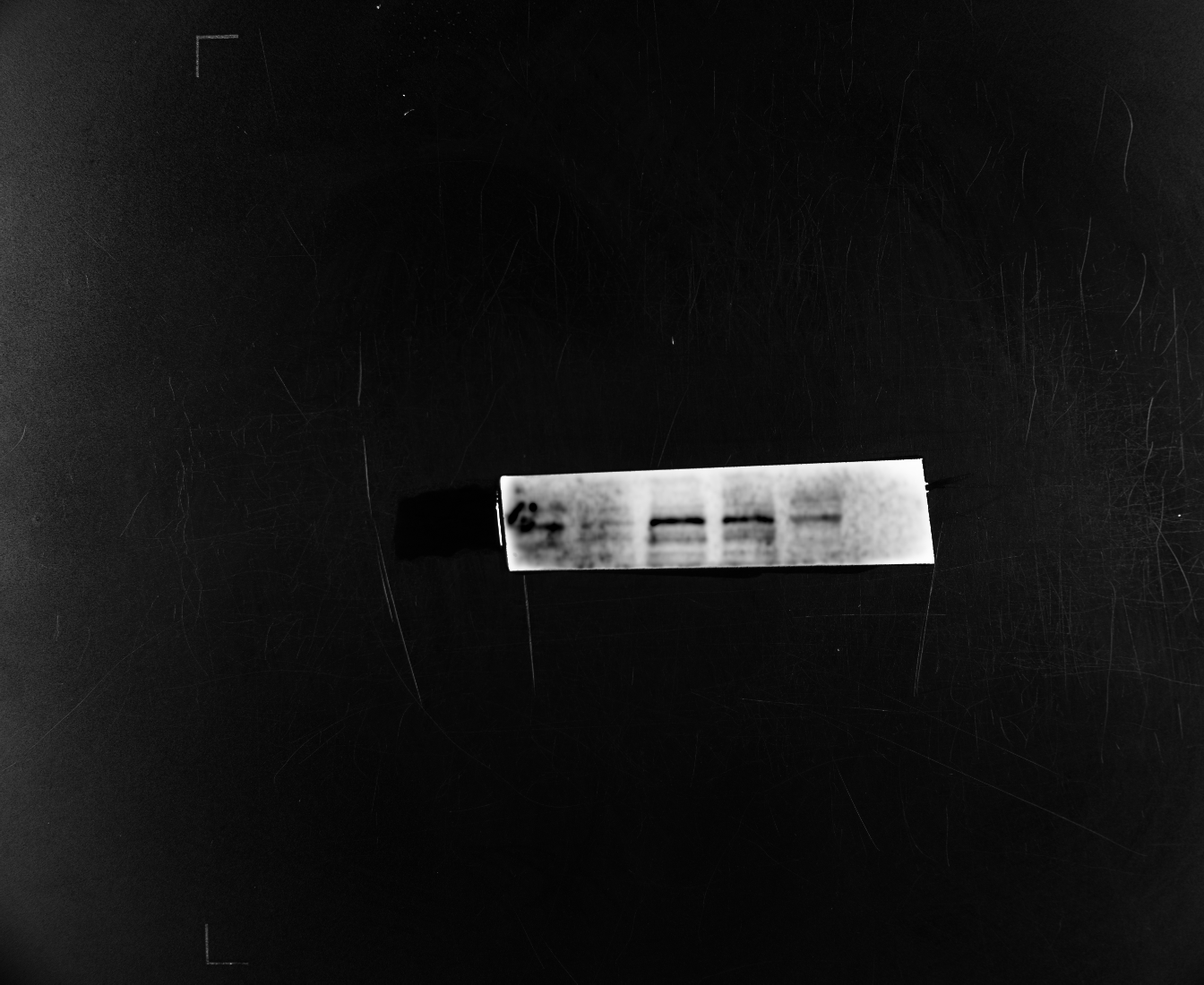


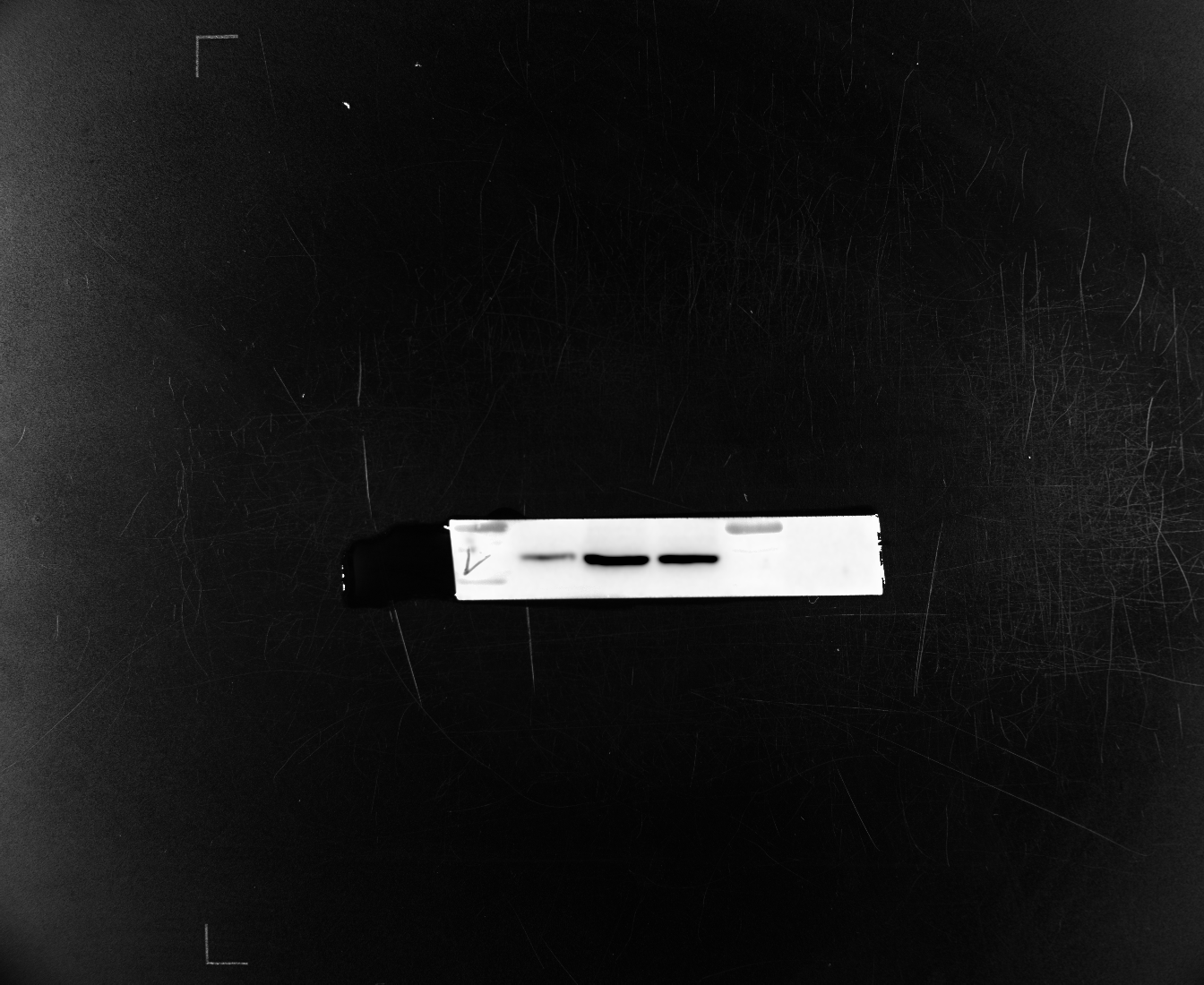


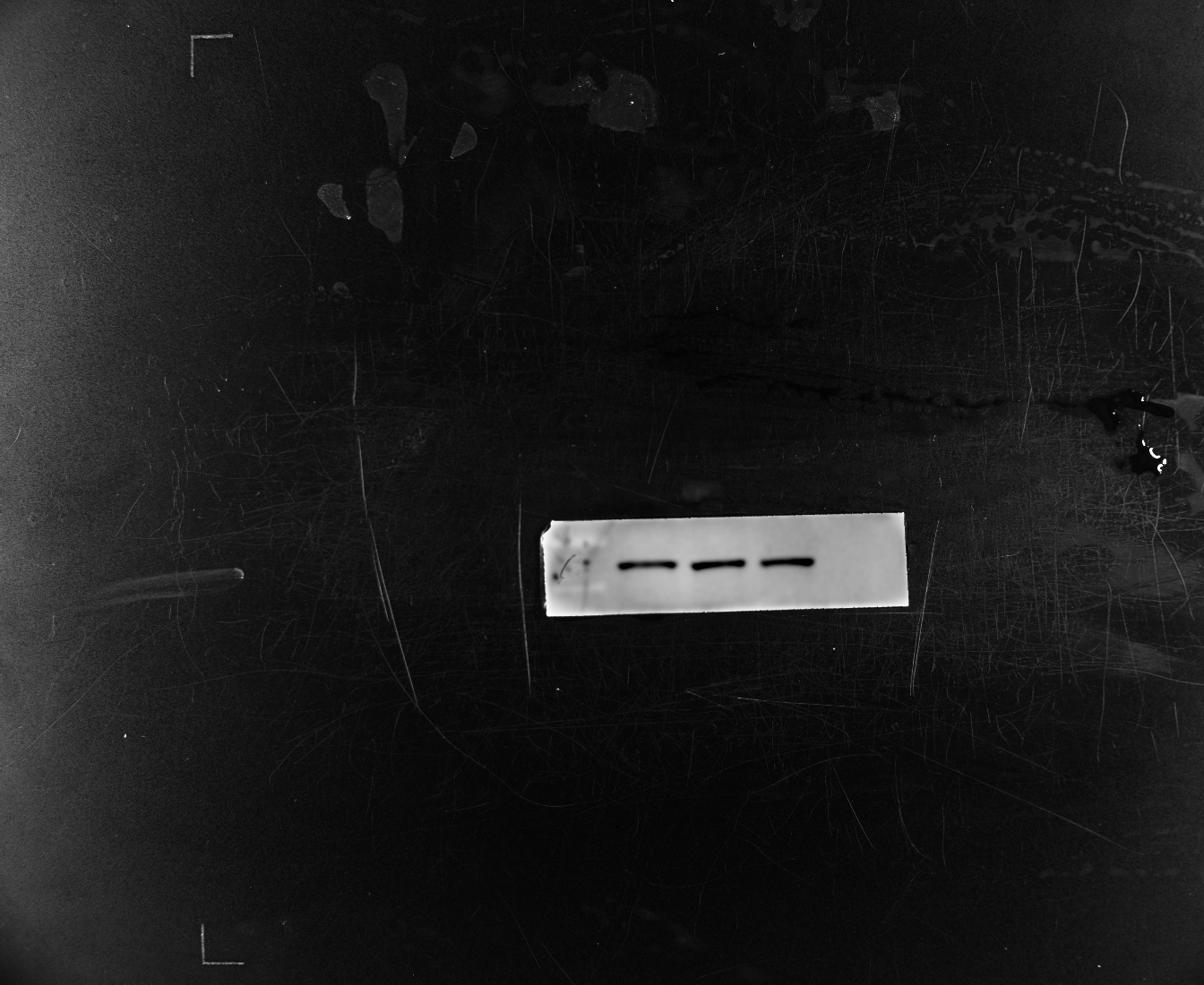


Figure 4D, E, F, G


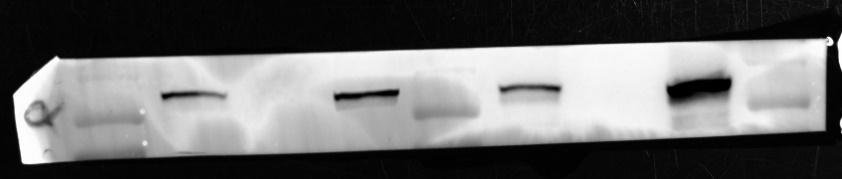


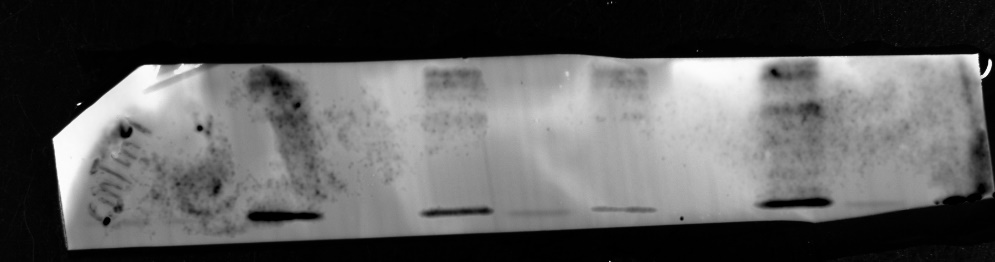


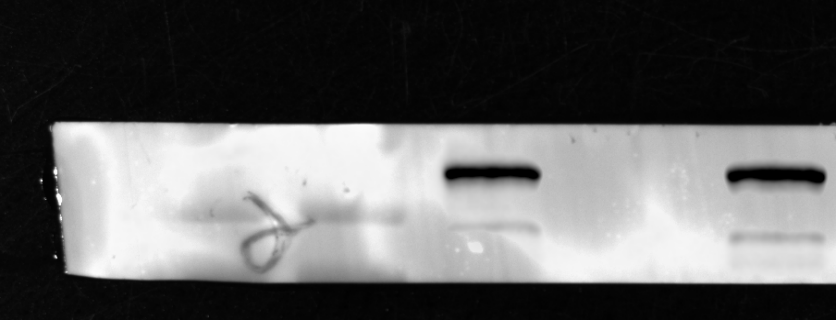


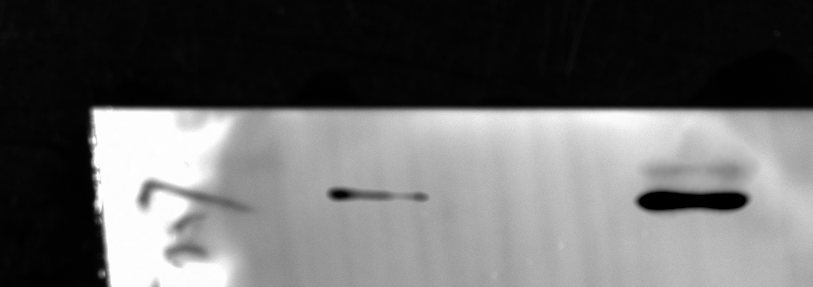


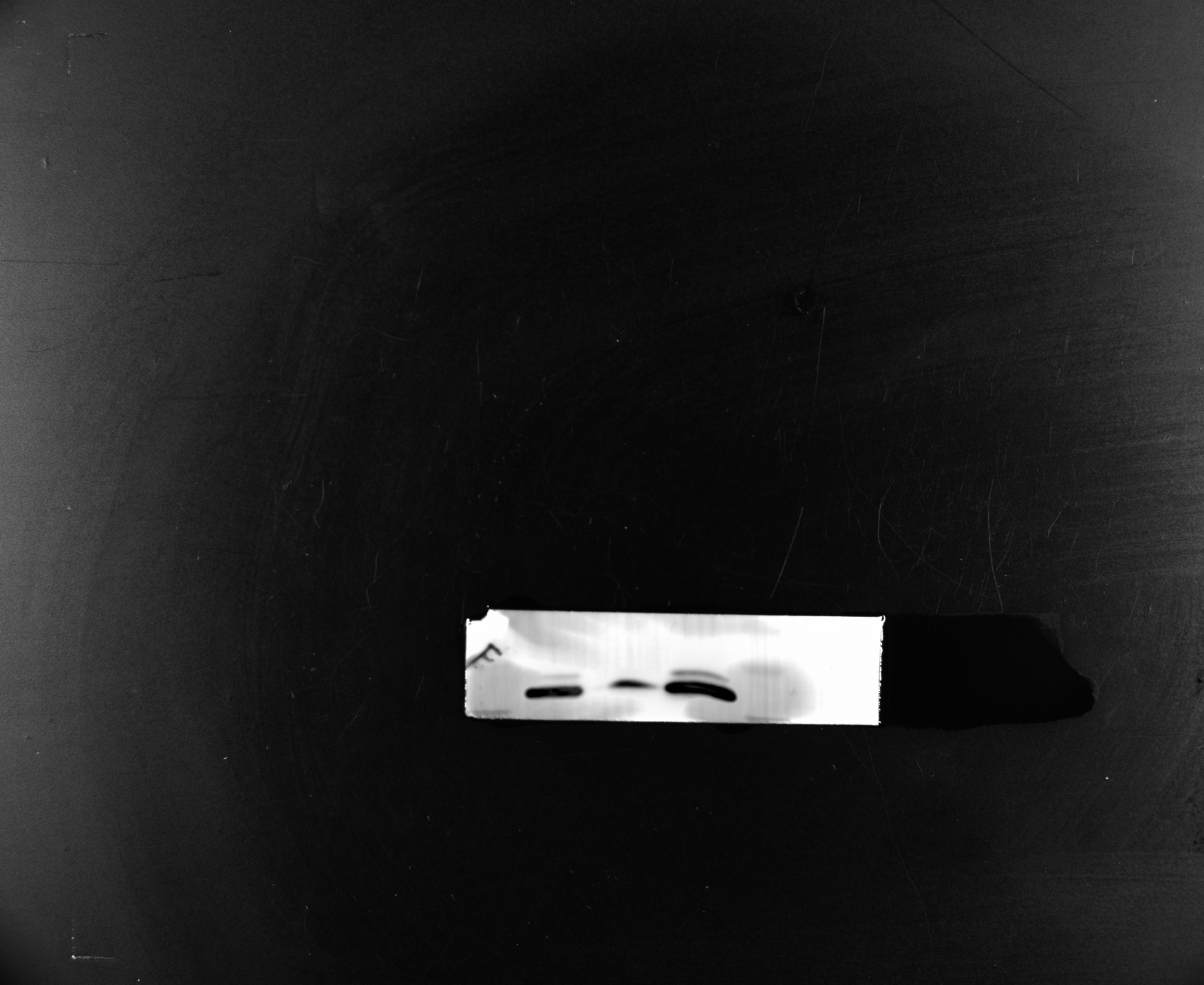


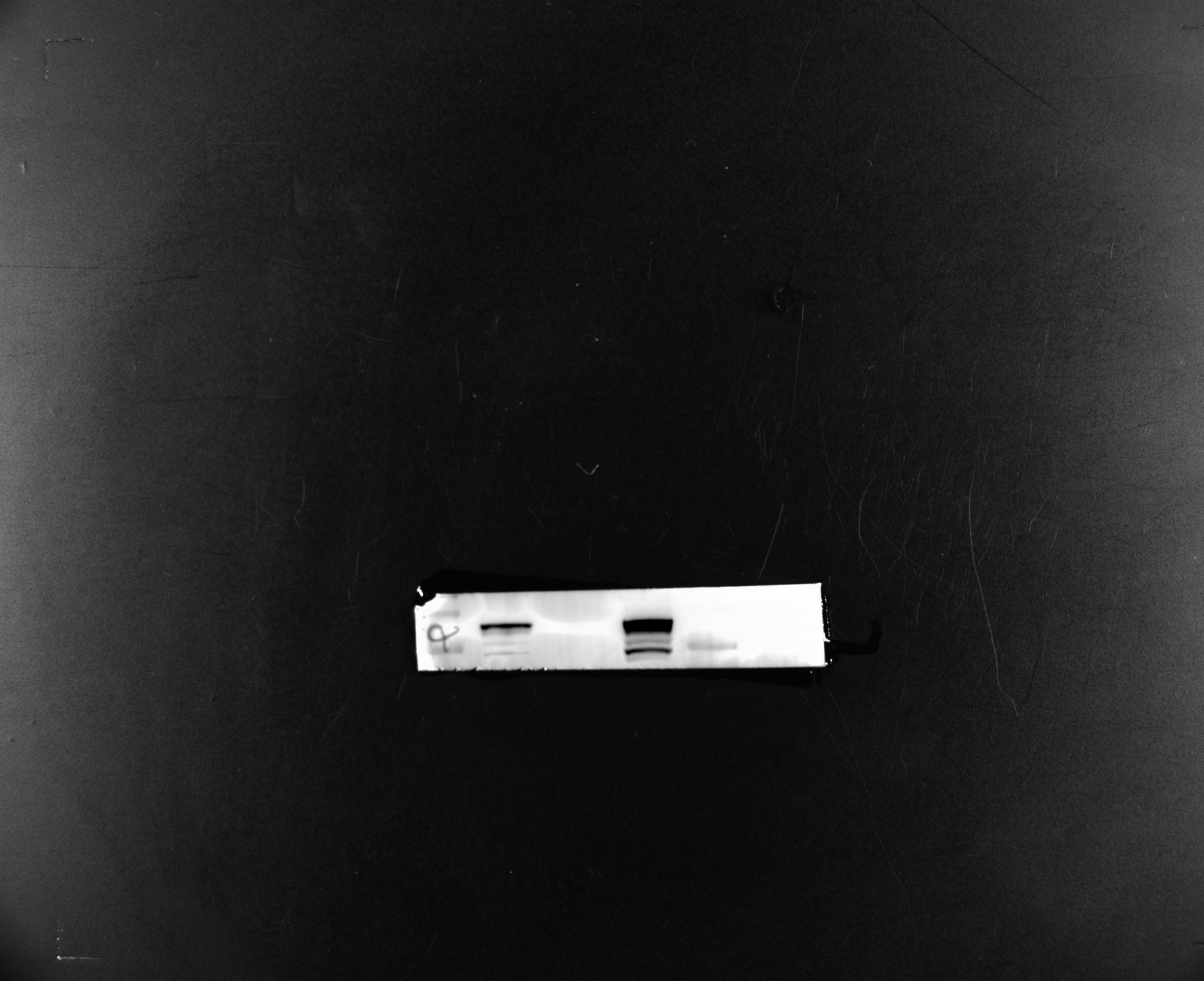


Figure 5E


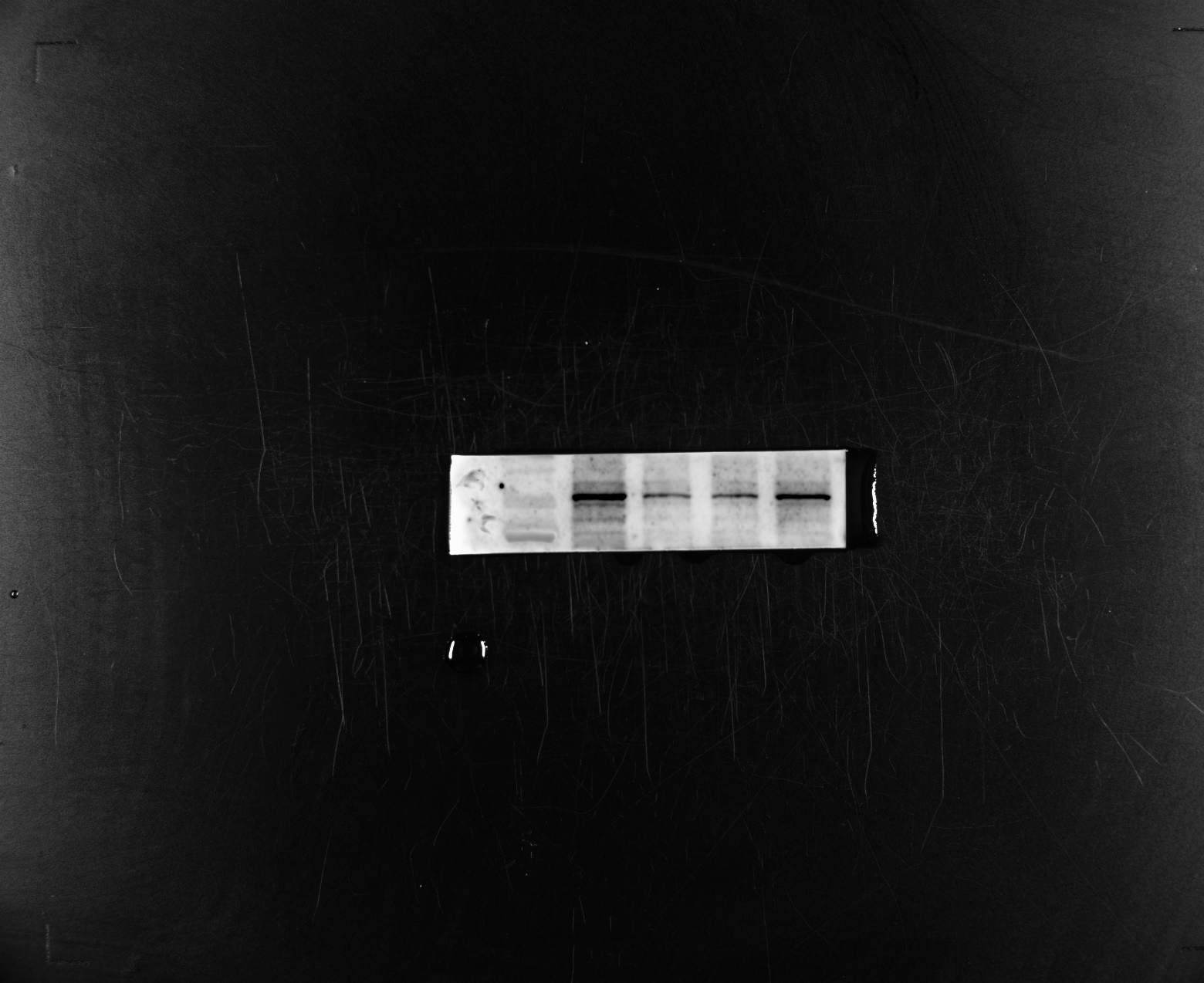


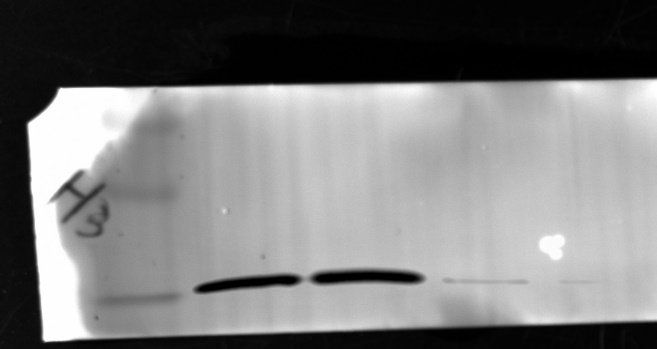


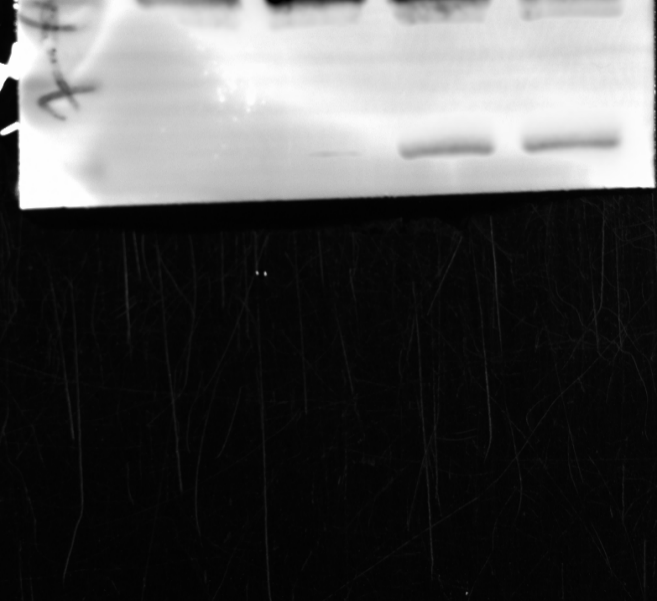


Figure 5F


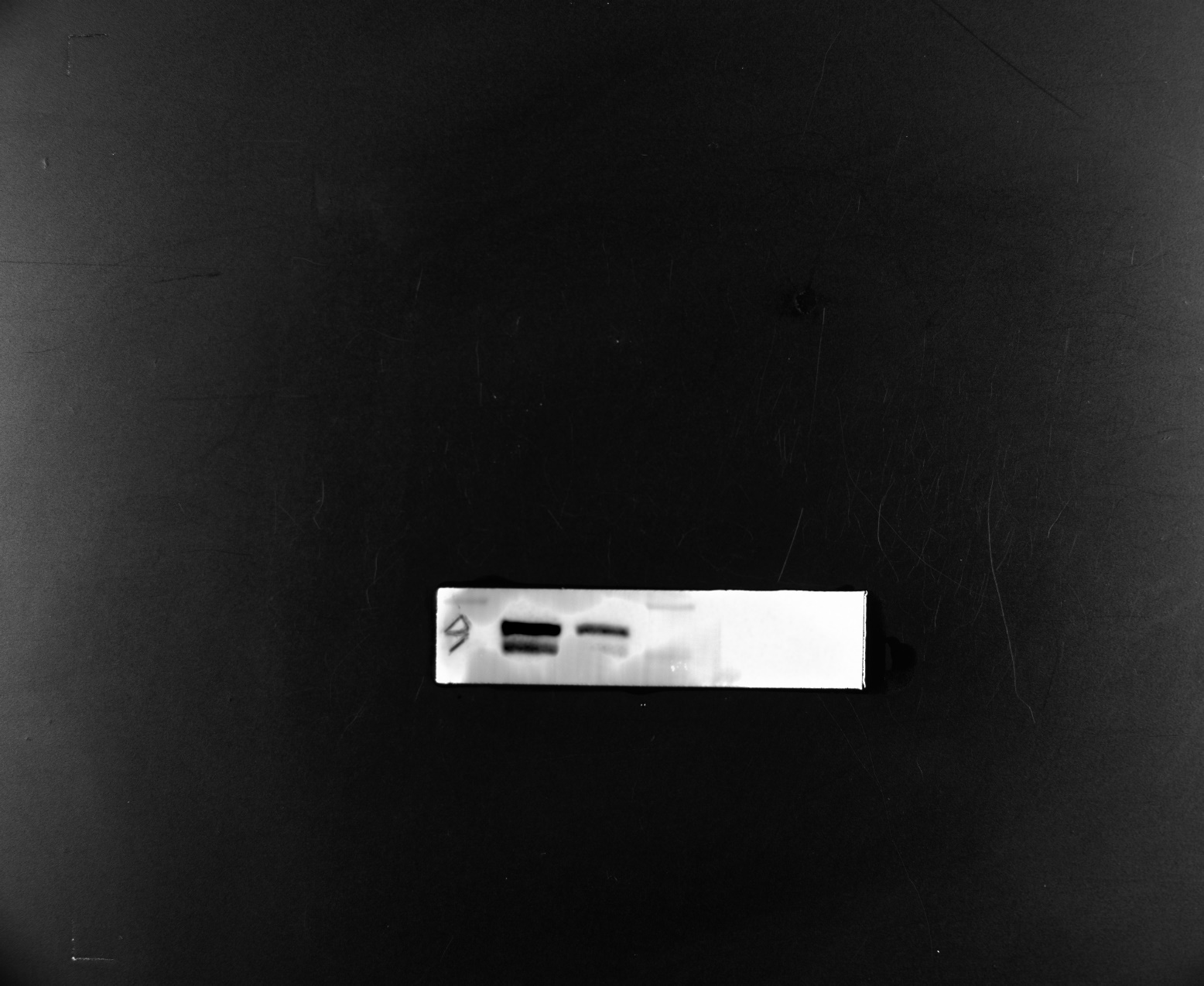


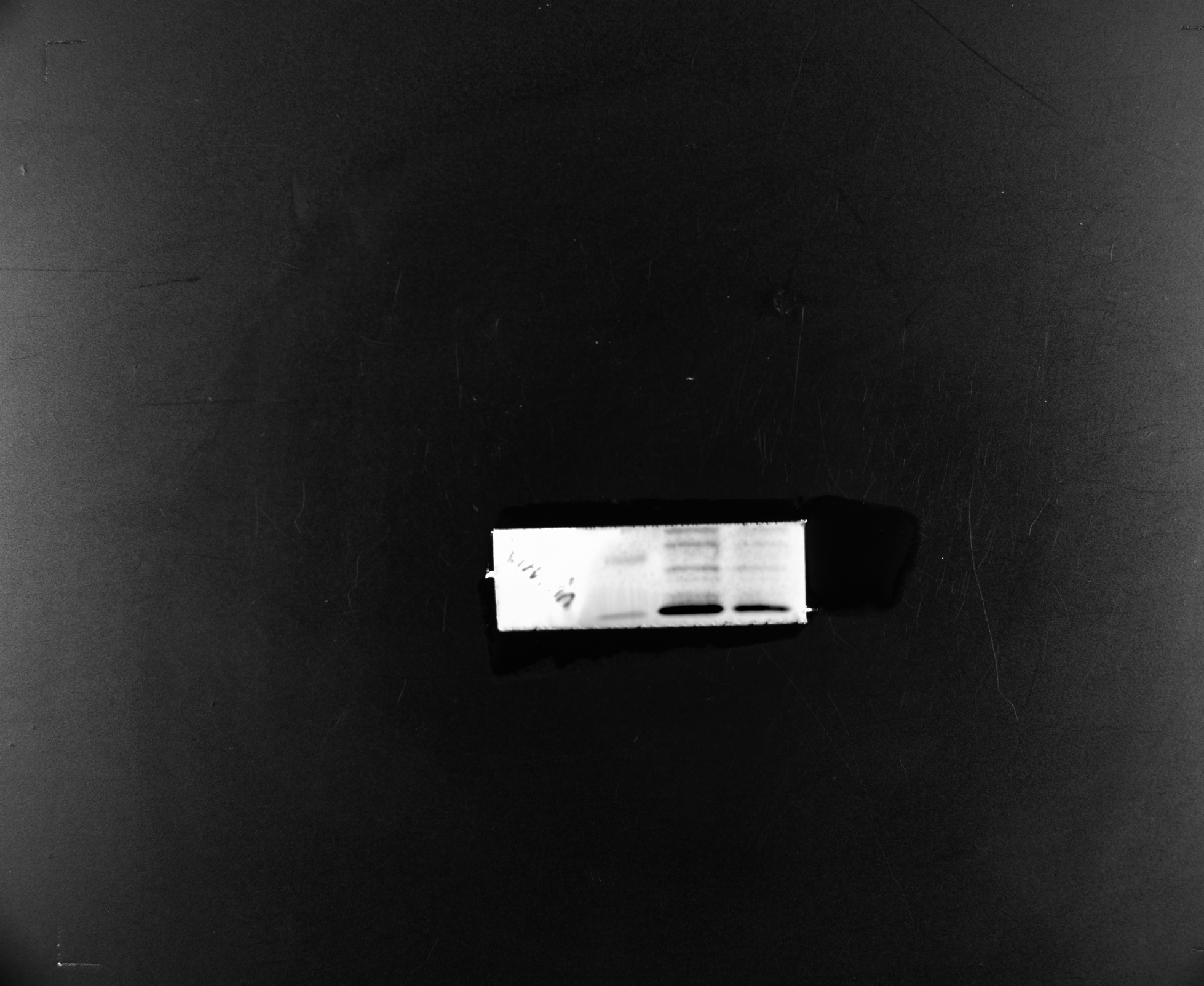


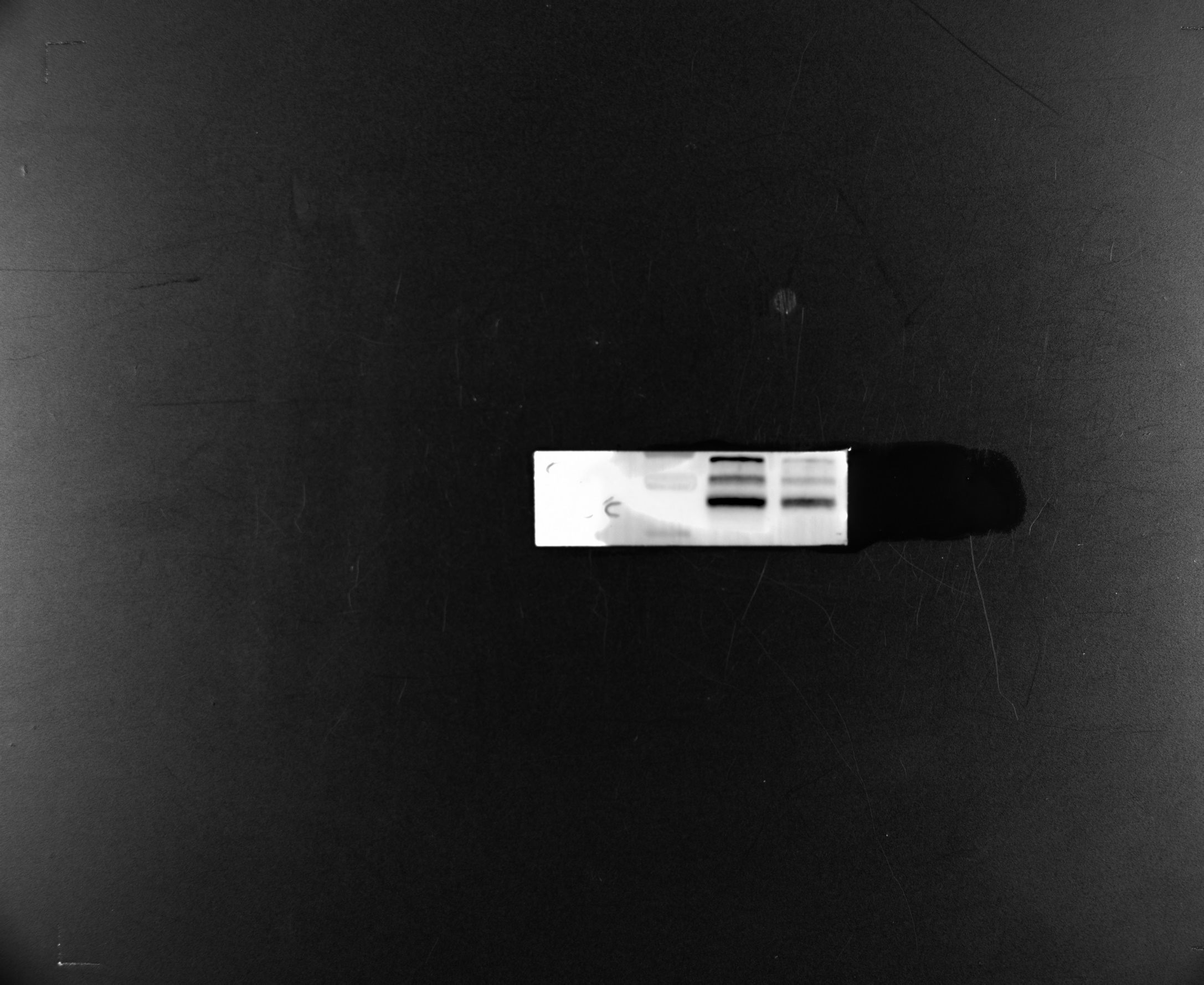


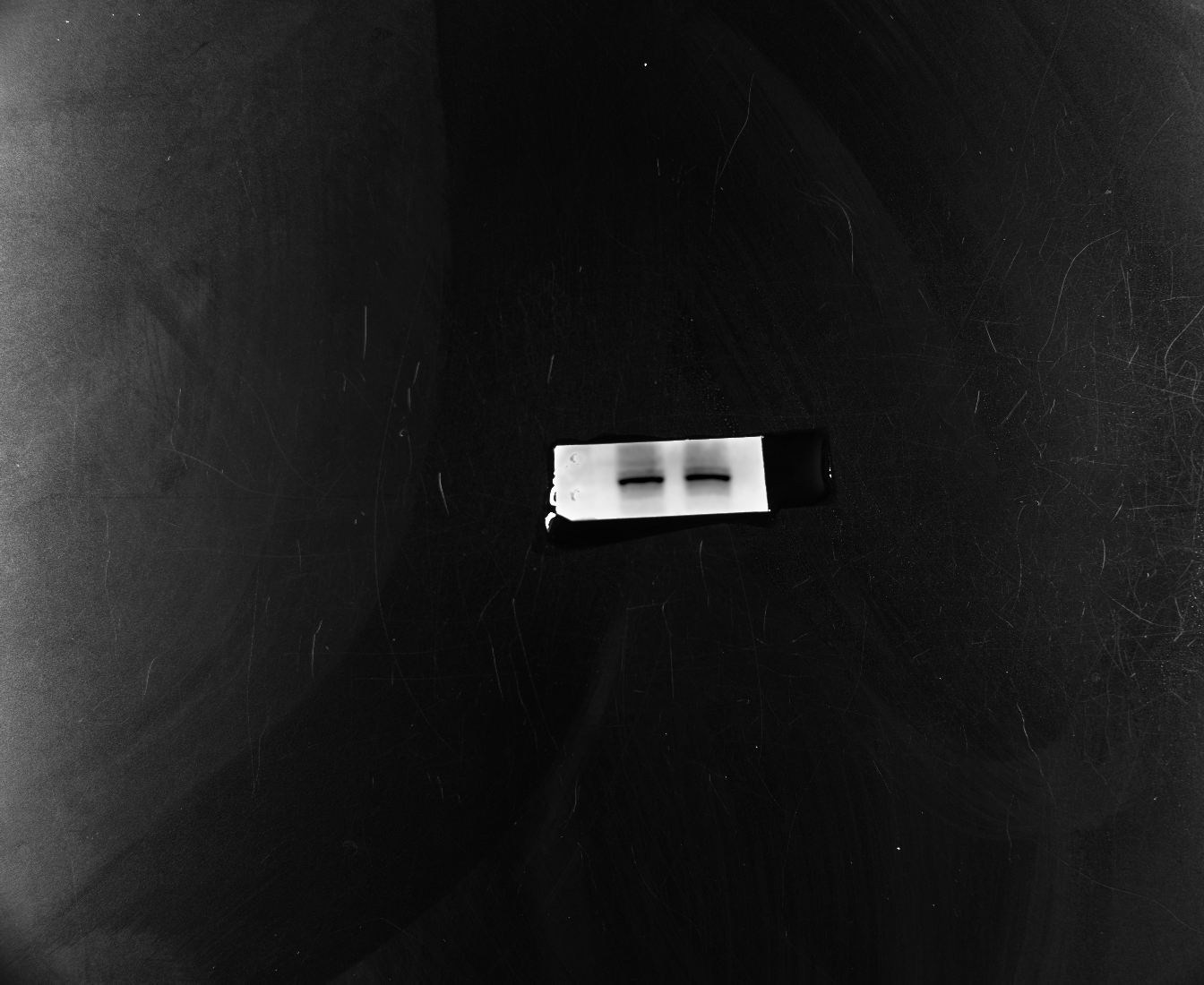


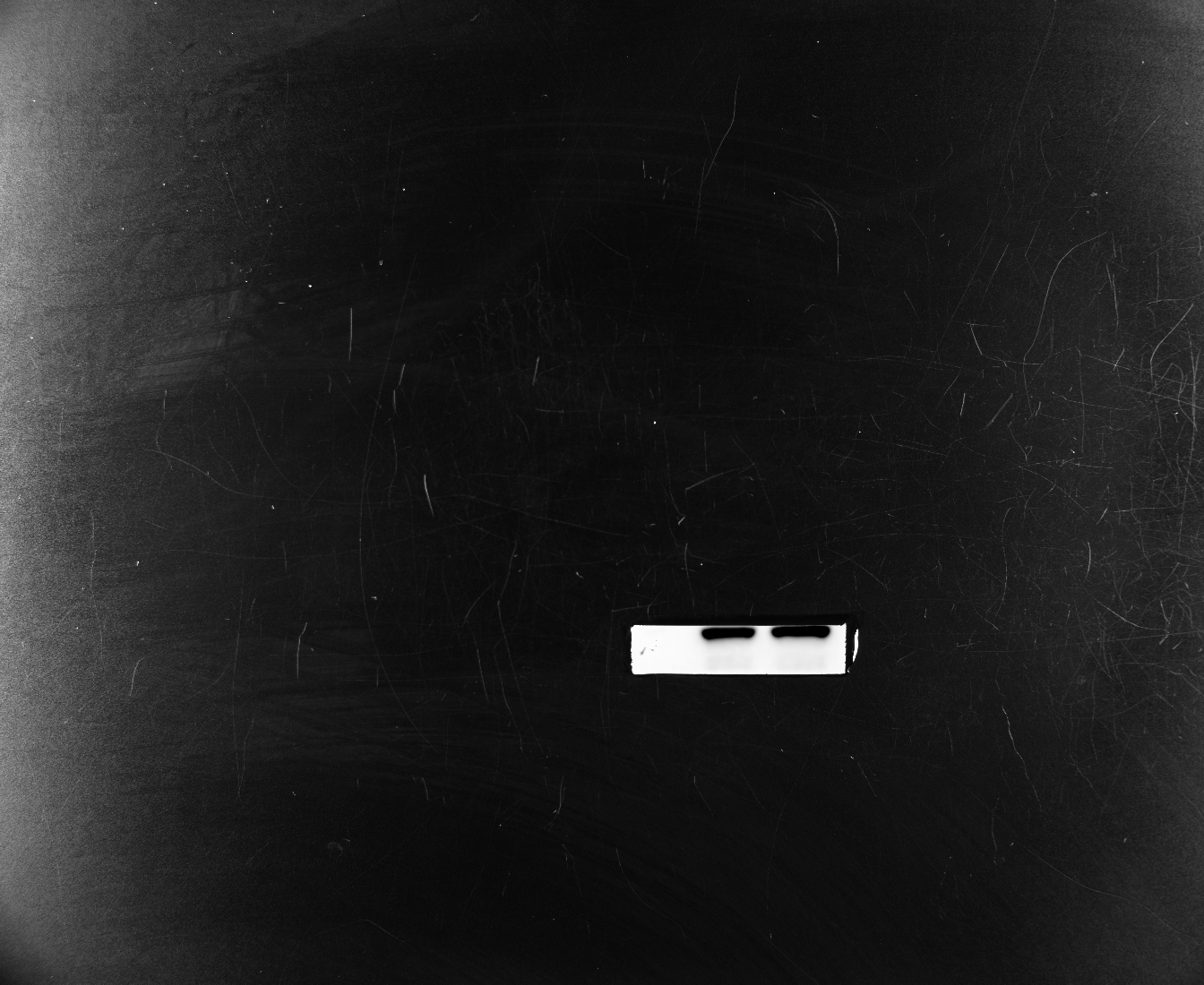


Figure 5G


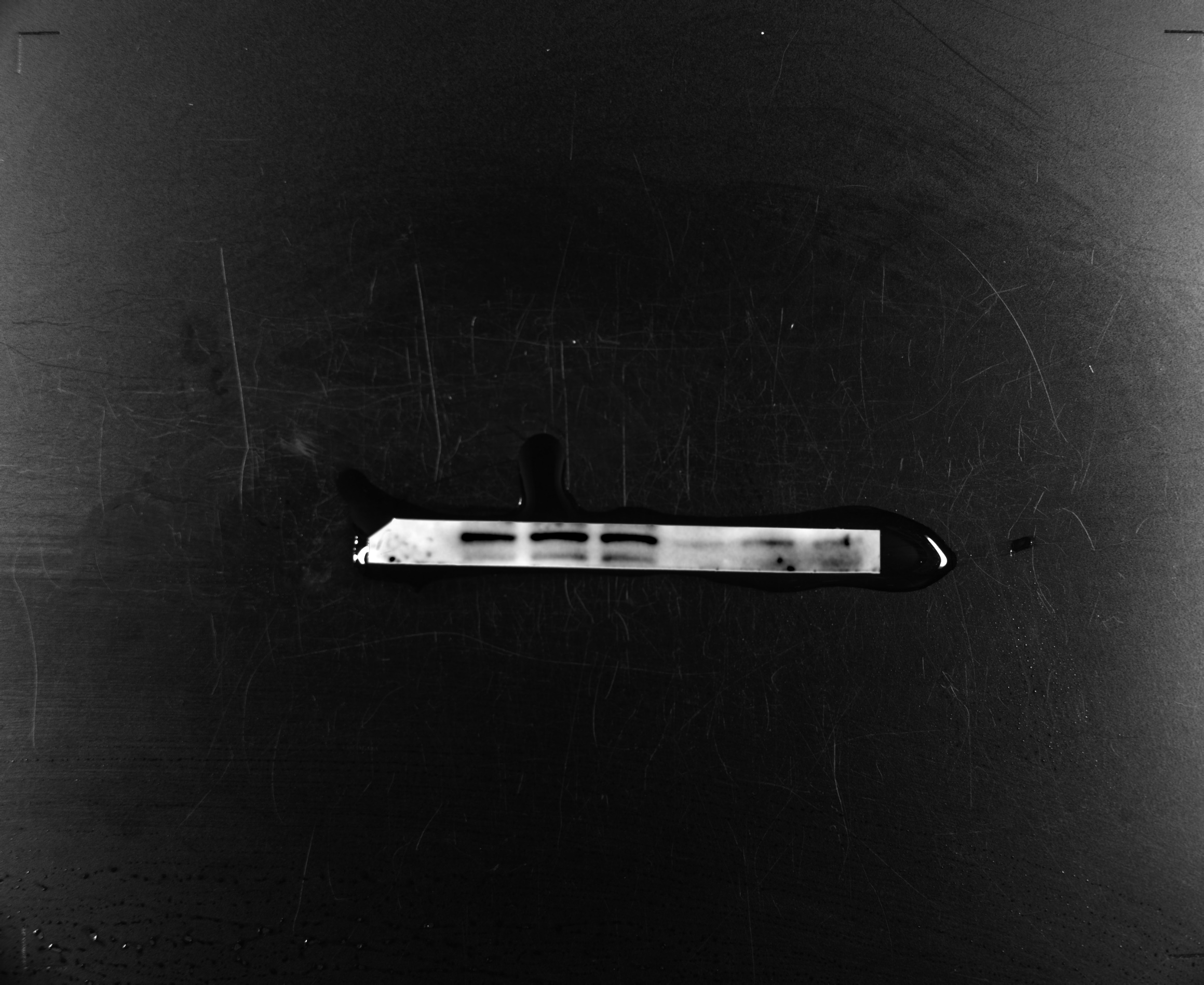


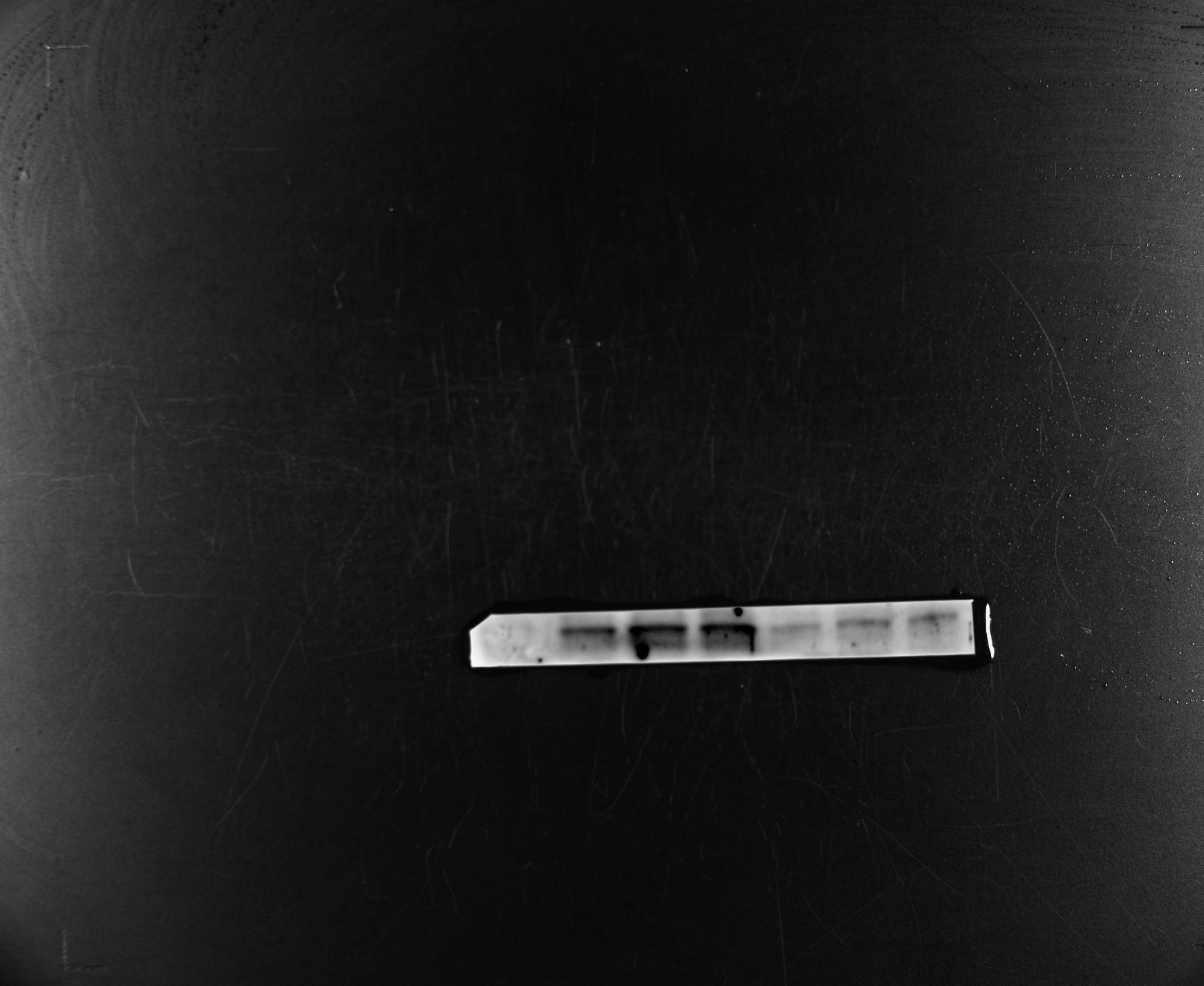


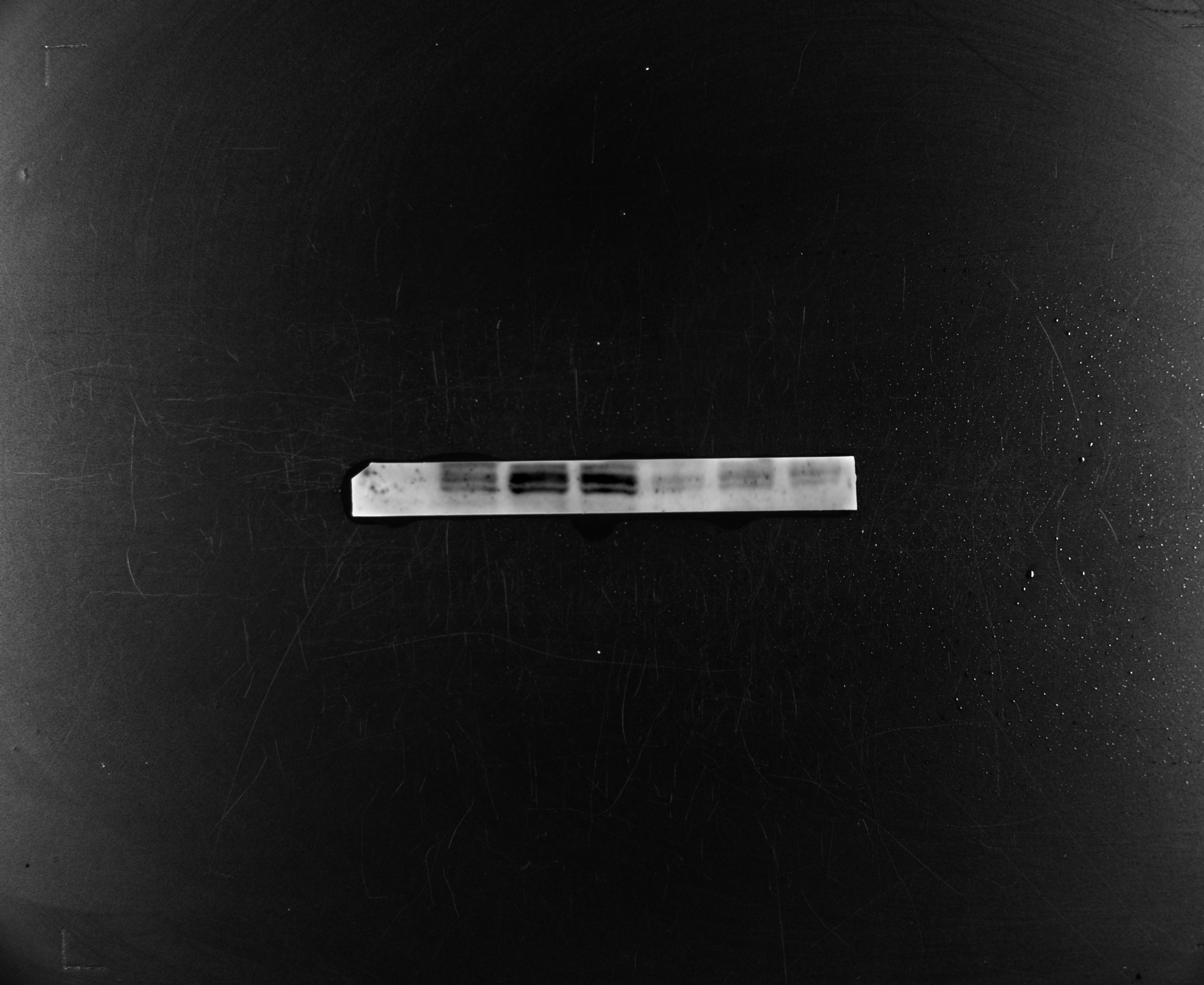


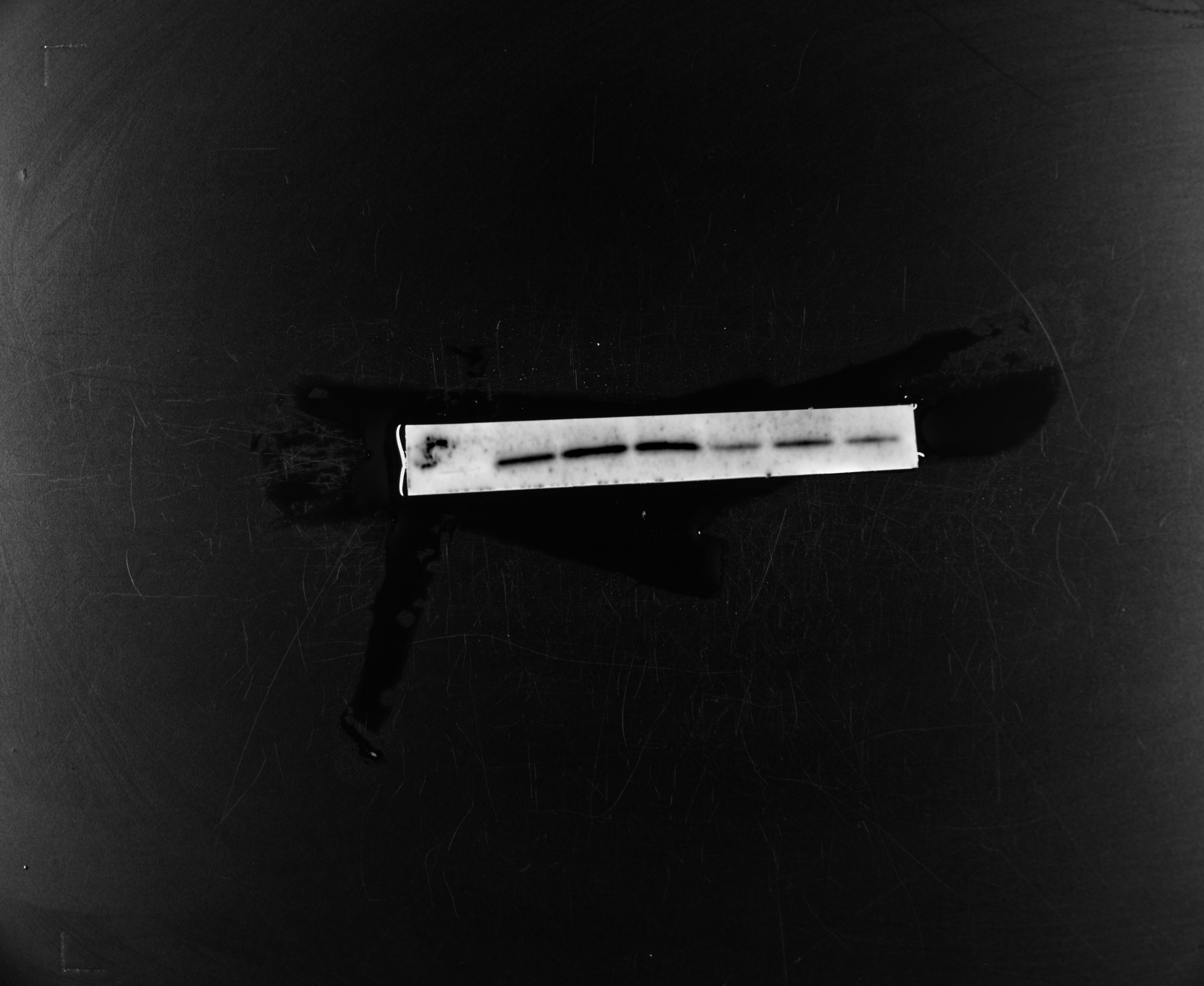


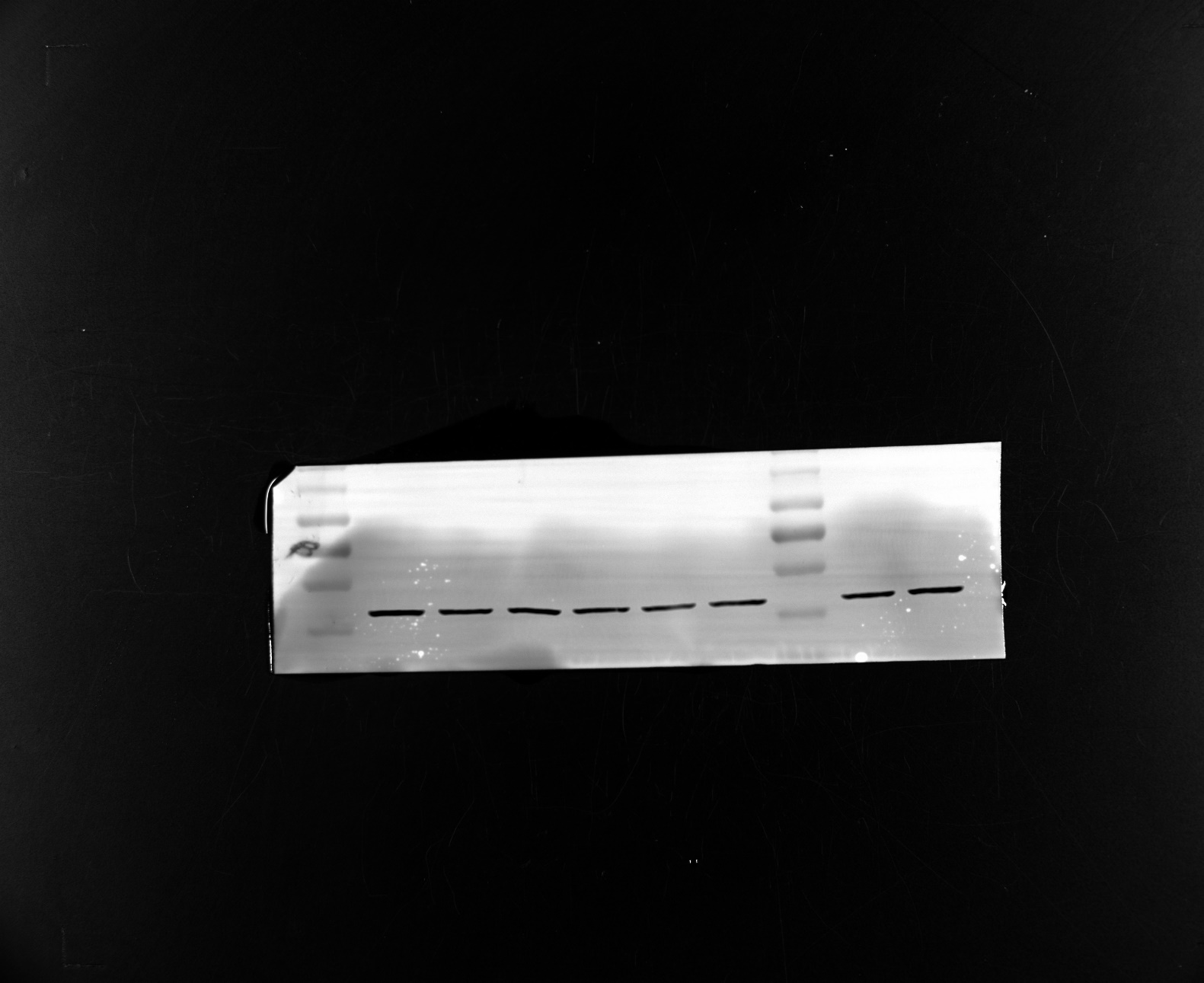


Figure 6G


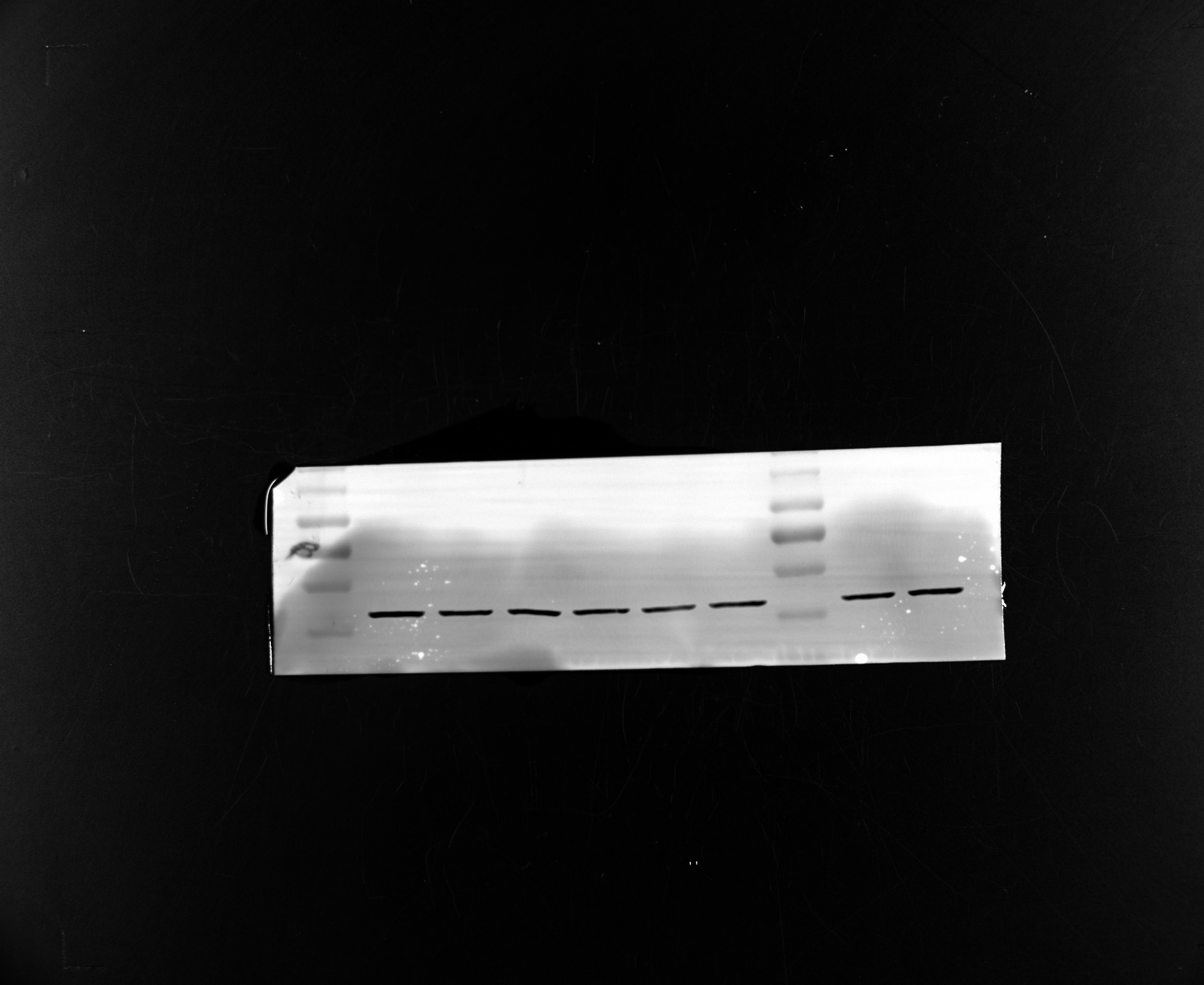


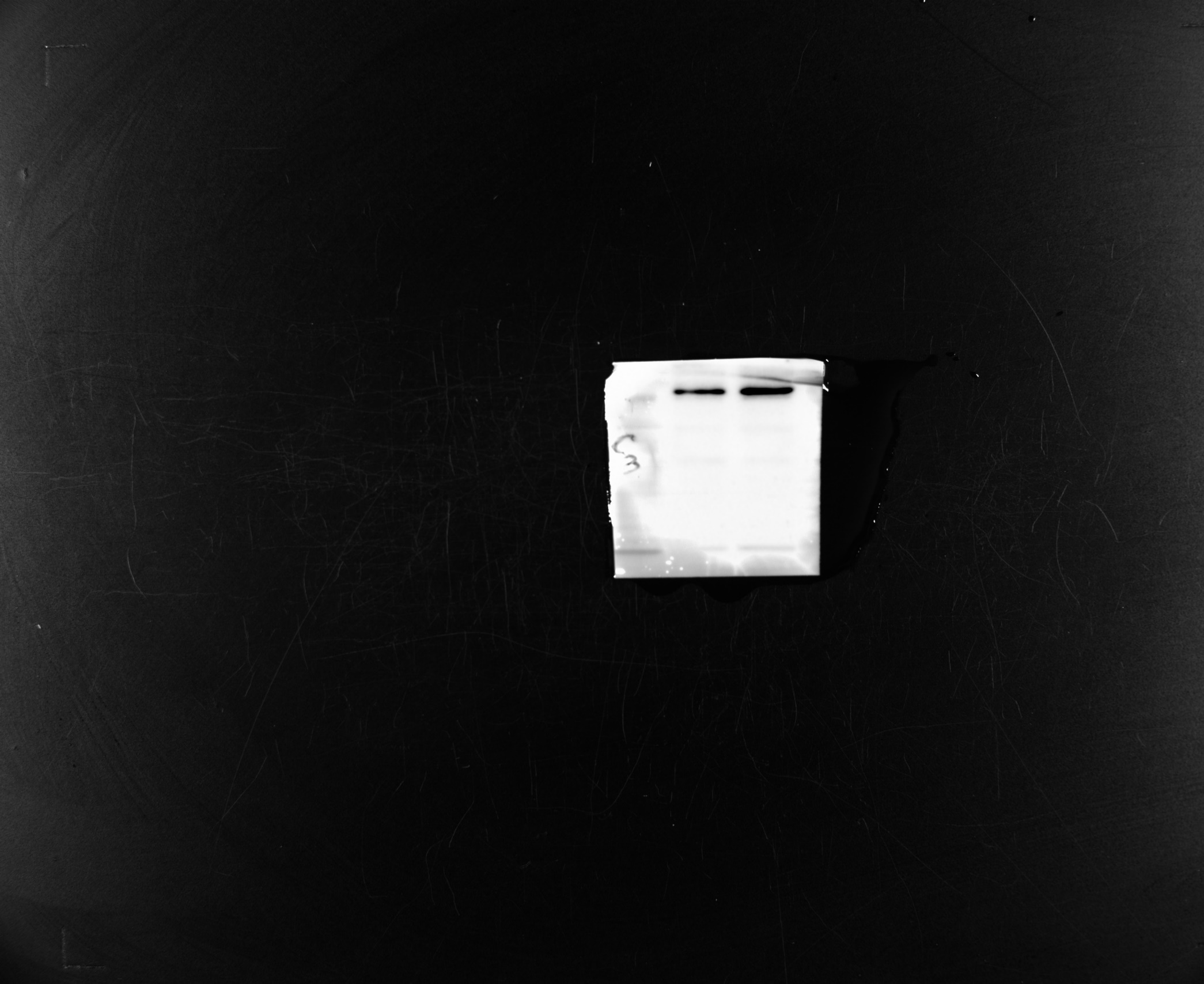


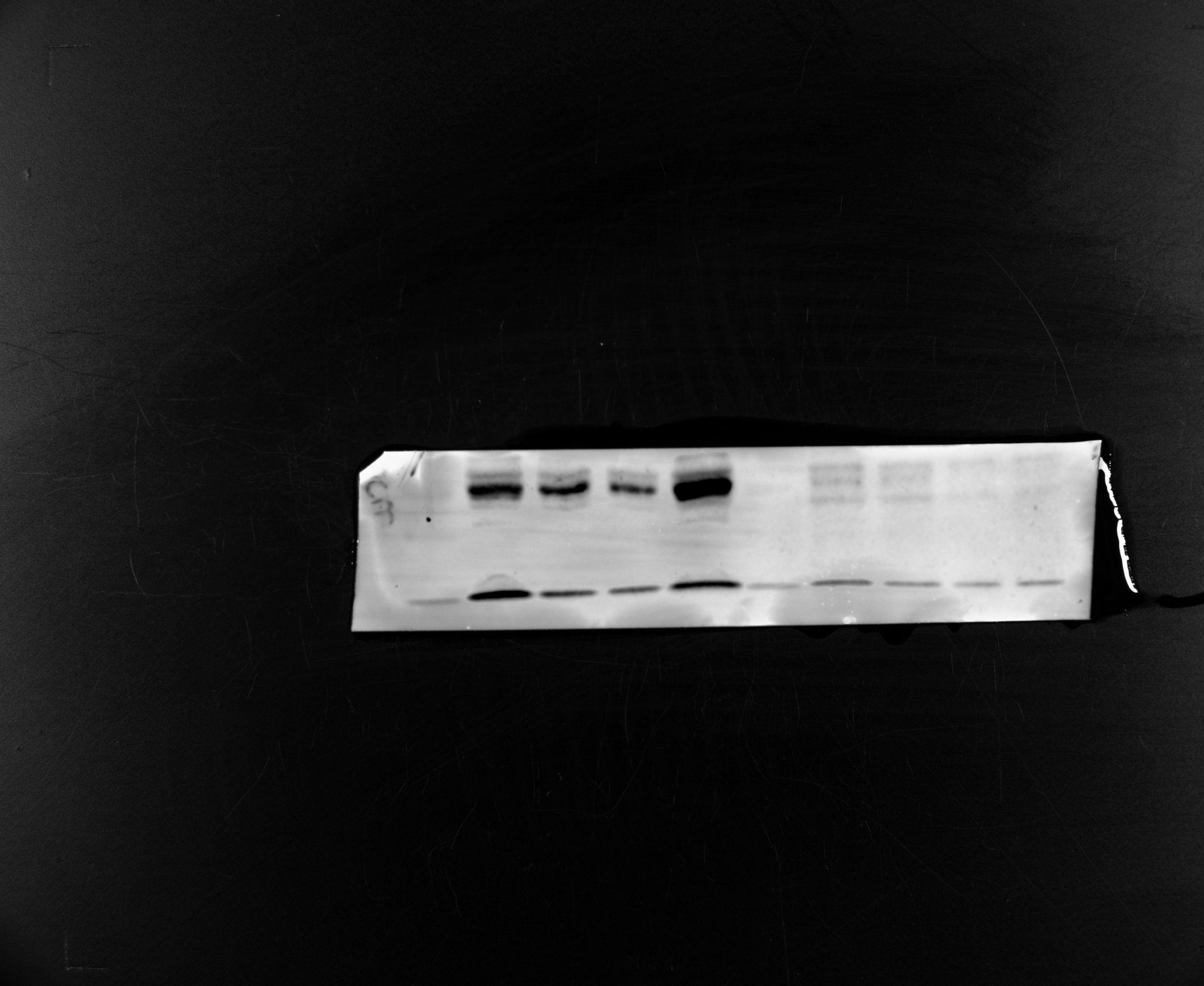


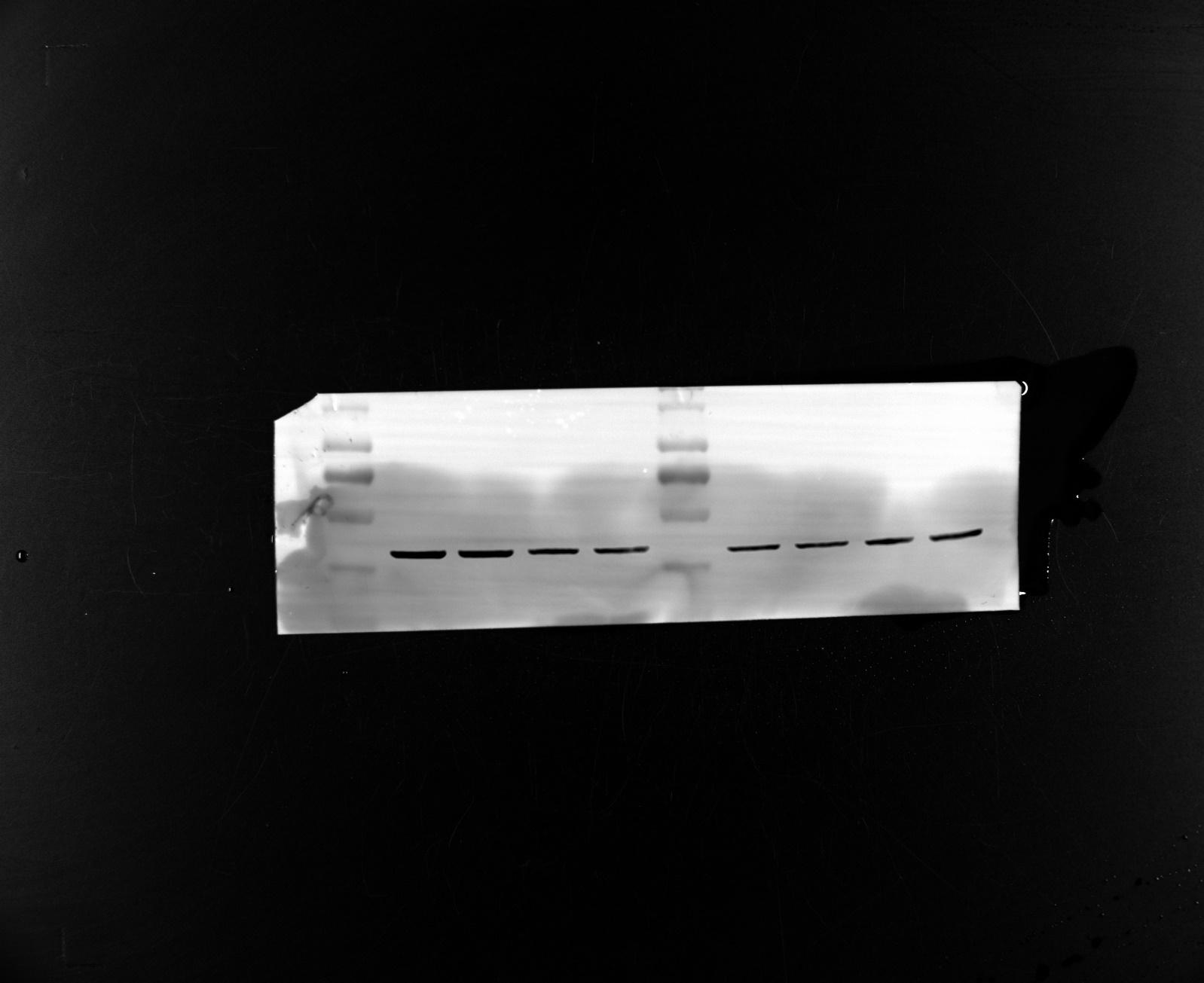


Figure 7H


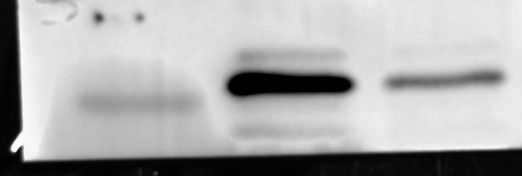


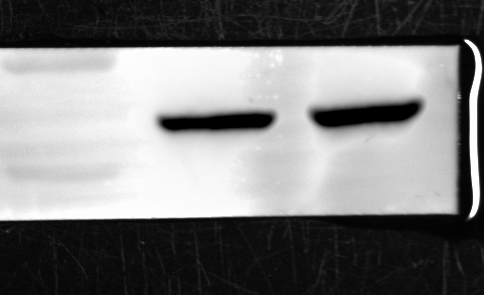


Figure 7J


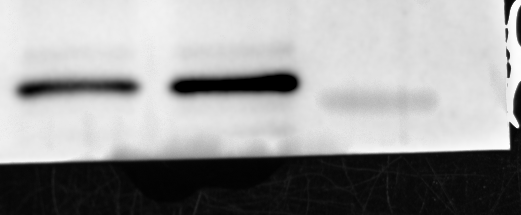


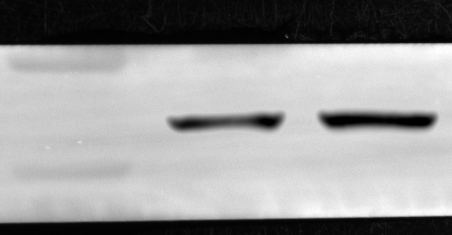


Figure S3 A


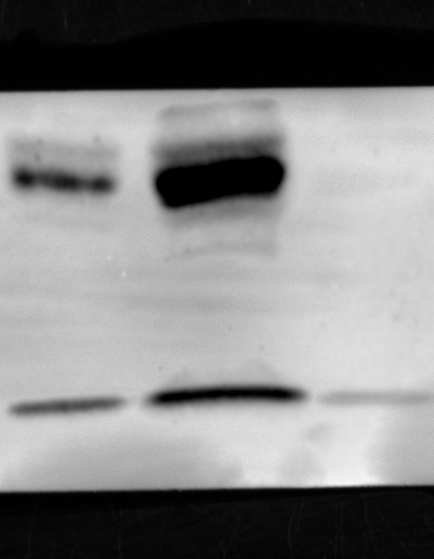


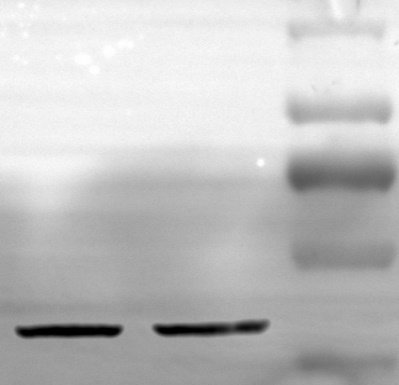


Figure S4 A


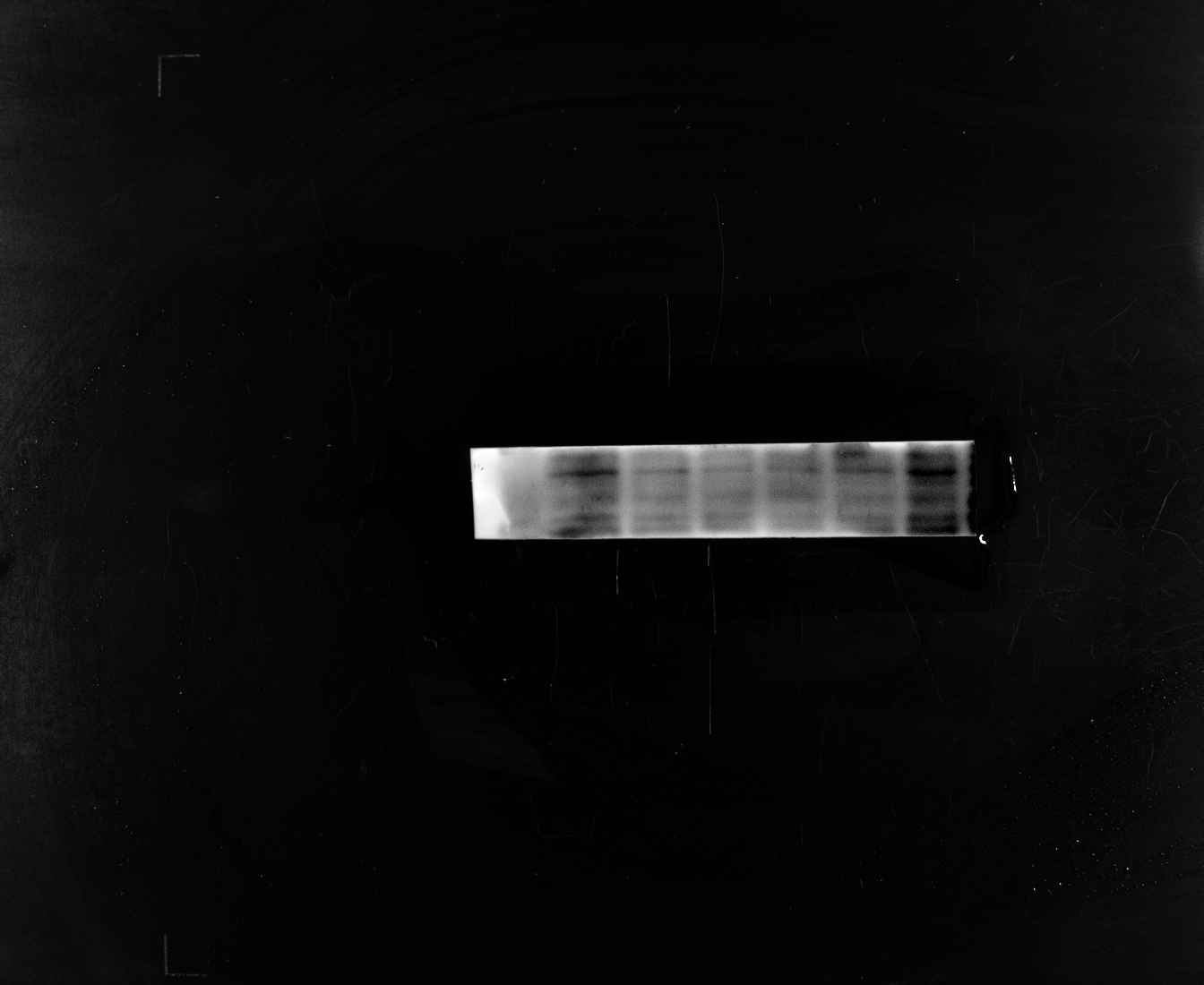


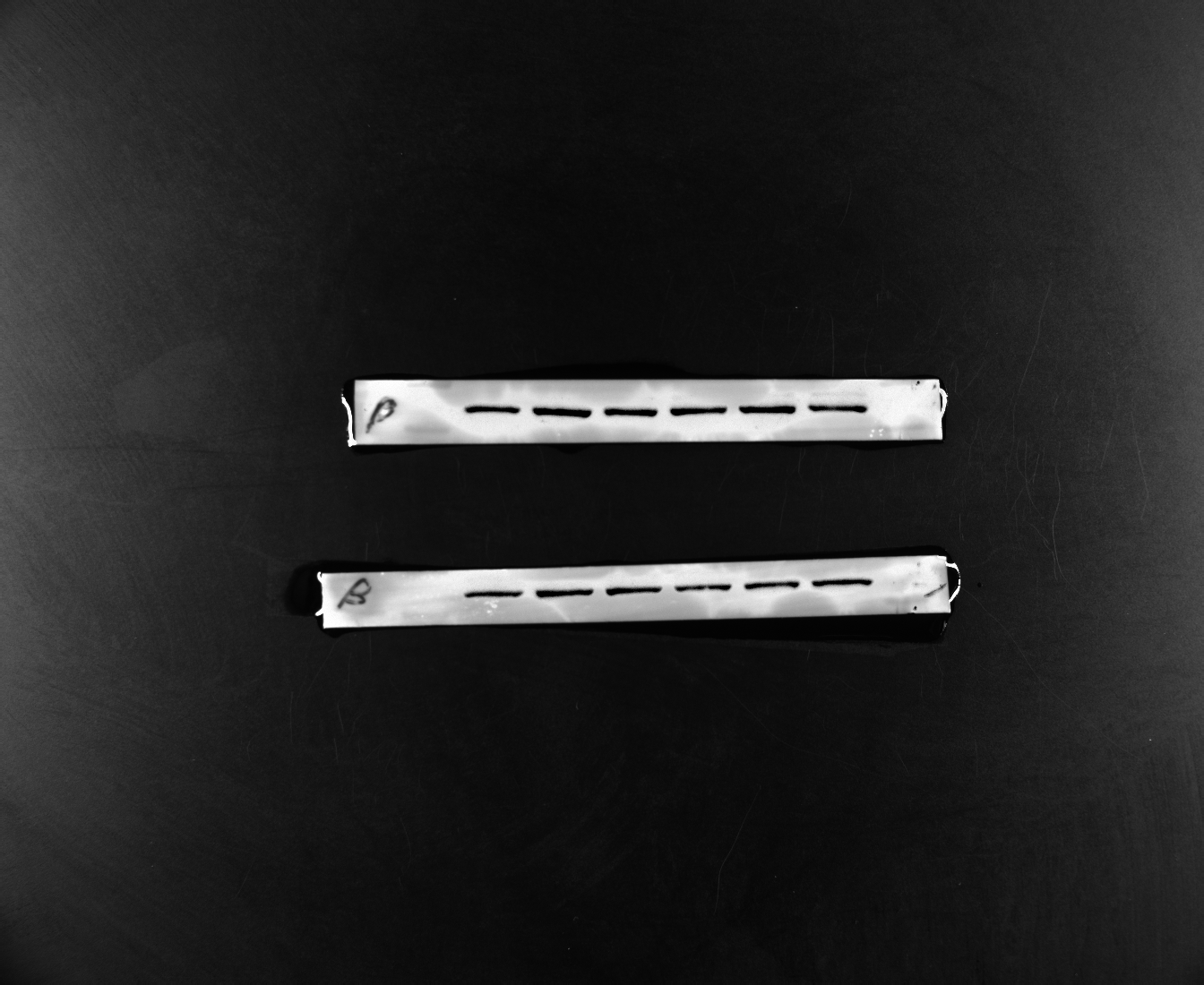


Figure S5 A


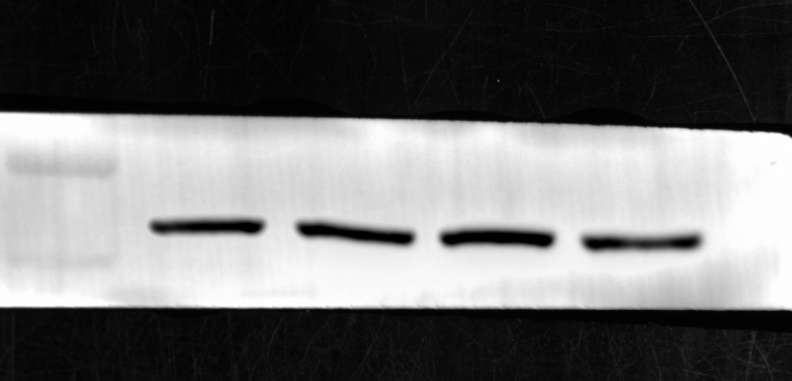


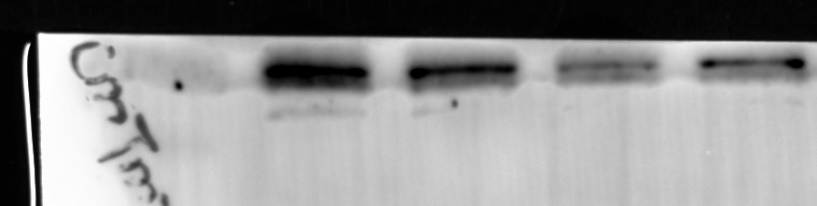

Supplement: Supplementary file 1 — Additional file 1. Supplementary tables and figures. [file 13058_2023_1620_MOESM1_ESM.docx]
